# Does Social and Organizational Support Moderate Emotional Intelligence Training Effectiveness?

**DOI:** 10.3390/bs14040276

**Published:** 2024-03-26

**Authors:** Ishara Madhunika Opatha, Yoshi Takahashi

**Affiliations:** Graduate School of Humanities and Social Sciences, Hiroshima University, Higashihiiroshima 739-8529, Japan; d212441@hiroshima-u.ac.jp

**Keywords:** training effectiveness, learning, training transfer, emotional intelligence, social and organizational support, organizational citizenship behavior, counterproductive work behavior

## Abstract

Given the dearth of systematic research and inconclusive results regarding the effectiveness of emotional intelligence (EI) training in adult training, this study was conducted to evaluate the effectiveness of EI training. This study aimed to assess the effectiveness of EI training on learning and transfer outcomes, considering underexplored moderation of social and organizational support with experimental and longitudinal research design. Training transfer was measured through changes in organizational citizenship behavior (OCB) and counterproductive work behavior (CWB). Participants self-assessed their OCB and CWB levels, while their supervisors also provided evaluations, allowing for separate analysis. Data, from a sample comprising 176 government officials, were collected across different periods and analyzed employing diverse analytical tools. The results revealed positive effects of EI training on training outcomes in both samples but positive moderation effect of social and organizational support on the effect of EI training on training outcomes was observed in the self-evaluation sample but not in the supervisor evaluation sample. The findings advance the debate on social exchange theory and organizational support theory by showing the boundary condition of their applicability. Furthermore, this study clarifies the impact of EI training on training outcomes by emphasizing the nuanced role of social and organizational support.

## 1. Introduction

In today’s rapidly changing and highly competitive world, organizations are increasingly recognizing the paramount significance of emotional intelligence (EI) in enhancing individual and organizational performance [1,2,3]. EI, characterized by individuals’ cognitive ability to recognize, understand, and regulate emotions both in oneself and others, has garnered significant attention as a crucial factor shaping various facets of organizational performance, such as improved communication [4], enhanced leadership effectiveness [5], increased job satisfaction [6,7], organizational learning [6], organizational commitment [8], organizational citizenship behavior (OCB) [9], counterproductive work behavior (CWB) [10,11], and better conflict resolution [12,13]. Therefore, in contemporary organizational settings, there is considerable focus on investing in EI training initiatives to cultivate and foster EI competencies among employees to improve organizational performance [2,3,14]. Despite substantial investments by organizations, a systematic investigation of EI training effectiveness has yet to be conducted to ensure the optimal allocation of resources in EI training [3,14,15,16,17]. This gap is primarily because of the lack of empirical studies that explicitly address the effectiveness of EI training programs in the workplace, which is further compounded by contradictory evidence from previous studies [3,14,18,19]. Consequently, some studies have pointed out that there are no comprehensive and systematic analyses to determine whether EI training interventions can be effective for adults in enhancing their EI [2,3,14]. Hence, addressing this gap, we were motivated to evaluate the effectiveness of EI training in the current study.

Training effectiveness is the degree to which a training program accomplishes its intended objectives and yields the desired outcomes by enhancing participants’ knowledge, skills, and performance [20]. This hinges on the trainee’s contentment with the improvement of their learning in terms of knowledge, skills, and attitudes (KSAs) [21] and their ability to transfer newly acquired learning to their work environment, leading to substantial changes [22]. Learning refers to the degree to which participants change their attitudes, enhance knowledge, and/or develop skills following program participation [23]. Training transfer is the degree to which trainees effectively utilize KSAs gained in the training context of their jobs [24,25]. Some studies have recommended evaluating the potential directions of learning and training transfer for future research [26,27]. Further, Baldwin and Ford’s model [28] provides a comprehensive framework for evaluating training effectiveness, focusing on learning outcomes and training transfer. Moreover, Kirkpatrick [29] emphasized that learning and transfer are interconnected and that learning is a vital factor in behavioral change. Based on these recommendations, we have selected learning and training transfer as the primary outcomes for assessing the effectiveness of EI training. 

Some studies have shown that EI training positively influences learning by comparing pre- and post-test EI scores [30,31,32,33]. Nevertheless, prior endeavors have failed to find a statistically noteworthy distinction between participants’ pre- and post-test scores [34,35], also presenting mixed evidence. Therefore, given these inconclusive results, analyzing the effect of EI training on learning can meaningfully contribute to the body of literature.

Evaluation of training transfer stands as a crucial indicator of training effectiveness, as noted by Leach and Li [36], and is “widely recognized as an important arena for research and practice” [36] (p. 42). Furthermore, an essential aspect of assessing training transfer is the evaluation of behavioral changes [37], as behavioral modifications influenced by training increase the likelihood of applying newly acquired KSAs to a work setting [38]. Reportedly, 40% of the acquired skills are immediately transferred, 25% persist for six months, and only 15% remain for a year [39]. Hence, understanding and improving training transfer remain ongoing concerns in the organizational context [27,40,41,42]. Moreover, the extant literature reveals that limited attention has been paid to training transfer owing to the challenges associated with implementation, complexity, cost, and the absence of direct evaluation approaches [43,44,45,46].

When contemplating the EI training transfer in our study, we identify OCB (voluntary helpful workplace behaviors that go beyond the strict requirements of the job role) and CWB (voluntary harmful workplace behaviors that go beyond the strict requirements of the job role) as the most suitable components to gauge behavioral changes. Miao et al. [47] suggested that it is important to understand the role of EI in OCB and CWB since EI was found to be one of the most vital predictors of OCB and CWB [47] because individuals high in EI regulate their emotions to excel at work and perform empathic prosocial behaviors such as increased OCB and decreased CWB but n [48,49,50]. Moreover, the existing literature predominantly focuses on evaluating the influence of inherent EI traits and abilities on OCB and CWB [47,50,51], and we observed a distinct scarcity of empirical studies investigating the impact of EI training interventions on changes in OCB and CWB. Consequently, our study endeavors to bridge this void by investigating the impact of EI training on changes in OCB and CWB.

Moreover, although some studies have found learning to be an antecedent of training transfer [52,53], only a limited number of studies have examined the mediating role of learning in the relationship between inputs in training and transfer [54,55]. Furthermore, there is a notable lack of empirical research investigating the mediating role of learning in the relationship between EI training and training transfer. Therefore, this study explored how learning serves as a mediator in the relationship between training and transfer.

Some studies have identified that work environment factors play a crucial role in comprehending the training transfer process [28,39,56,57,58,59,60]. Richman-Hirsch [61] stressed that the potential boundary conditions of the work environment in the relationship between training and transfer have not been investigated in previous studies. 

Social and organizational support is considered a vital factor in the work environment and should be designed to effectively transfer acquired KSAs to the workplace [26,28,39,59,60,61]. Furthermore, drawing from the principles of social exchange theory (SET) and organizational support theory (OST) [28,62,63,64,65], we argue that when social and organizational support is high, employees who participate in training try their best to acquire new KSAs and transfer them to the workplace more so than those with a low level of social and organizational support. We believe that the moderating role of social and organizational support in terms of EI training effectiveness has been understudied. Hence, we investigated how social and organizational support moderates the effectiveness of EI training in terms of learning and training transfer outcomes.

When assessing the effectiveness of EI training programs, an apparent gap in research is notable in training interventions, particularly in the domain of transfer, since most studies primarily concentrated on the results of pre- and post-tests to demonstrate the change among a single group of participants and failed to provide comprehensive details of EI interventions and process development [2,66]. Therefore, Farnia and Nafukho [2] recommended that future research on EI training evaluation should prioritize experimental and longitudinal studies since such studies could make a substantial contribution to the literature on EI development. 

Accordingly, this study aimed to evaluate the effectiveness of EI training on training outcomes, considering the underexplored moderation of social and organizational support, through a randomized controlled trial (RCT) and employing a longitudinal design.

## 2. Literature Review and Hypothesis Development

### 2.1. Key Concept Definitions and Theoretical Foundations

#### 2.1.1. Emotional Intelligence

EI has gained significant prominence as an emerging research focus within psychological, organizational, and educational disciplines [67]. Salovey and Mayer [68] proposed the foundational theory of EI. Salovey and Mayer [68] specified EI as “the subset of social intelligence that involves the ability to monitor one’s own and other’s feelings and emotions, to discriminate among them and to use this information to guide one’s thinking and actions” (p. 189).

The skills, traits, and abilities associated with EI are understood and explained through various theoretical concepts and models [69,70,71,72]. Three prominent theoretical models play crucial roles in shaping EI research: the ability model of Mayer and Salovey [72], the trait model of Petrides and Furnham [73], and the mixed model [69,70,74].

According to the ability model, EI is conceptualized as a set of specific emotional skills and abilities with four dimensions: perceiving emotions, facilitating thoughts using emotions, understanding emotions, and managing emotions [72,75]. Researchers who view EI as an ability largely agree that it encompasses a collection of behaviors and skills that significantly influence an individual’s ability to recognize and effectively manage their own emotions, as well as those of others [3]. Mayer and Salovey introduced the Mayer–Salovey–Caruso Emotional Intelligence Test (MSCEIT), a widely recognized instrument for evaluating EI. Individuals can use this tool to assess their EI proficiency across multiple domains, including perception, utilization, comprehension, and management, enabling them to comprehensively understand their emotional competencies.

Petrides and Furnham [73] introduced the EI trait model. They proposed that EI comprises a collection of personality traits or emotionally linked dispositions rather than cognitive abilities. This model emphasizes that individuals possess inherent emotional personality traits that determine their behaviors, including perceiving, managing, and responding to emotions [76]. 

Addressing the conceptual differences between the ability and trait models, both Bar-On’s social–emotional intelligence and Goleman’s EI competency models are considered mixed models [69,70,74]. The mixed model of EI combines a range of cognitive abilities and emotional dispositions [2,77] and encompasses other non-cognitive features, namely, social skills, motivation, self-esteem, and personality characteristics [78]. Goleman’s competency model is instrumental in assessing success in the workplace [70]. This model encompasses four key domains: (a) self-awareness, (b) social awareness, (c) self-management, and (d) relationship management [79]. This further highlights the fact that EI skills can be learned and developed.

Among EI models, we focused on the ability model because it facilitates the monitoring of skill development and establishes a link between training impact and noticeable workplace enhancements in terms of evaluating training effectiveness [80]. Unlike trait and mixed models such as self-report measures, the ability model employs an ability-based framework, providing a more objective assessment of individuals’ skills in perceiving, using, understanding, and managing emotions [81]. Therefore, the ability model contrasts with models that focus primarily on personality traits related to EI, ensuring a more direct and practical evaluation of actual skills and abilities in terms of measuring learning outcomes. In addition, many studies have employed the MSCEIT as a tool to gauge KSAs to assess the effectiveness of EI training programs on learning [30,82]. Moreover, the ability model is widely regarded as the most suitable model for characterizing the concept of EI and forecasting the outcomes of socially relevant behaviors, such as OCB and CWB [48,50], since high-ability EI employees can make effective use of emotion regulation mechanisms to foster positive expectations for social interactions, which results in positive citizenship behaviors [83]. Therefore, we adapted the ability model to evaluate the impact of EI training on learning and training transfer outcomes.

#### 2.1.2. Learning

Learning is identified “as the extent to which participants change attitudes, improve knowledge, and/or increase skill as a result of attending the program” [23] (p. 22). Baldwin and Ford’s model [28] suggests that training has a significant effect on learning. This theoretical framework highlights the acquisitions of KSAs during the training process. Evaluation at the learning level usually involves a formal test that gauges the KSA levels of the participants before and after training interventions. 

#### 2.1.3. Training Transfer, OCB, and CWB

Training transfer encompasses the procedure of transferring newly obtained KSAs from the training to the job and maintaining these KSAs over time [56]. Baldwin and Ford’s model [28] also emphasizes that training has a significant effect on transfer. In our study, we measured the changes in OCB and CWB to measure training transfer before, 1 month after, and 3 months after the training. OCB is defined as “not directly or explicitly recognized by the formal reward system, and that in the aggregate, promotes the efficient and effective functioning of the organization” [84] (p. 4). It refers to voluntary behavior that enhances organizational effectiveness, extending beyond employees’ formal job responsibilities or expected performance roles [85,86]. 

Meanwhile, CWB is defined as “the voluntary behavior of organizational members that violates significant organizational norms, and in doing so, threatens the well-being of the organization and/or its members” [87] (p. 356). Fox et al. [88] asserted that CWBs encompass negative behavior towards organizations and colleagues, causing serious organizational damage [89,90].

#### 2.1.4. Social and Organizational Support

It has been identified that the work environment plays a role in increasing or decreasing the transfer of KSAs obtained from training to the workplace. This phenomenon has been called “the transfer climate”, denoting elements perceived by trainees as either promoting or hindering the transfer of KSAs obtained from the training [91]. Richman-Hirsch [61] highlighted that previous studies dealing with training transfer did not examine how the work environment might moderate training transfer.

In the work environment, researchers have increasingly focused on social and organizational support in studies on transfer climate [62,92] because it plays a significant role in training transfer climate for determining the use of training in the workplace [93]. Nevertheless, we did not observe any studies on the moderating effects of social and organizational support on EI training effectiveness. Therefore, we selected social and organizational support to explore this moderating role.

Social support refers to trainees’ perceptions of their support for their work tasks [94]. It encompasses the trainee’s beliefs, level of concern, and appreciation exhibited by others at the workplace, and how much value they value their contribution to the workplace [59]. According to the extant literature, four dimensions of social support can be observed: supervisor support, peer support, supervisor sanctions, and feedback/coaching [95,96]. This study focused on evaluating social support with a specific emphasis on supervisor and peer support. Supervisor support is defined as the extent to which supervisors support and reinforce learning on the job [97] because they encourage, motivate, provide clear directions, and support their subordinates in transferring KSAs by removing obstacles [98,99]. Peer support refers to how much trainees perceive their colleagues or peers as supporting the use of the KSAs acquired from training at work [100,101,102,103]. 

Organizational support is defined as an employee’s perception of their organization as concerned with their well-being and valuing their contribution to the organization [65,104,105]. When employees believe that their organizations support their tasks, value their contributions, and prioritize their well-being, they tend to have positive attitudes and invest effort beyond their prescribed duties [106,107,108].

#### 2.1.5. Theories Related to Social and Organizational Support

##### Social Exchange Theory

SET is a fundamental framework in social psychology that offers an understanding of the dynamics of social relationships by examining patterns of exchange and interactions among individuals [109]. According to SET, when social support is readily available, employees are expected to repay their organizations by investing more effort in enhancing job performance [104,110], employee engagement [111], and employee commitment [104]. Based on SET, when employees perceive that their organization has demonstrated higher social support, they are more inclined to engage in behaviors that are beneficial to the organization, such as OCB enhancement and CWB decrement [104,112].

##### Organizational Support Theory

OST is a theoretical framework that explains employees’ concerns about the treatment they receive from an organization to extend their contribution to the organization [99]. By OST, employees develop perceptions regarding how their organization supports and values their contributions and demonstrates concern for their growth and well-being [65,104,113]. These can be manifested through organizational support, namely organizational rewards, job enrichment, promotions, relationships, fairness perceptions, concern for employees’ goals and values, and offering help when they want it [105]. Employees reciprocate by improving organizational performance, behavior, involvement, and commitment [114,115].

### 2.2. Hypothesis Development

#### 2.2.1. Emotional Intelligence Training on Learning

The literature presents mixed evidence regarding the impact of EI training on learning. Some studies have found a positive impact of EI training on training outcomes, including learning skills, abilities, and knowledge [3,116,117,118,119,120,121]. In addition, some experimental studies have demonstrated that EI training positively affects experimental groups. For example, in the study of Nelis et al. [30], a brief empirically derived EI training was administered only to experimental group members (four group sessions lasting 2.5 h) and the training design was based on Mayer and Salovey’s four-branch model. The results indicated that the experimental group showed notable improvements in emotion identification and emotion management abilities, whereas no significant changes were observed in the control group. Subsequently, Pool and Qualter [82] developed a course that included short lectures, video clips, case studies, group tasks, role plays, a trip to an art gallery, and reflection journals based on Mayer and Salovey’s [72] model of EI. Among the experimental group, results indicated a positive change in emotional self-efficacy regarding understanding and managing emotions. Vesely et al. [31] administered an intensive 5-week teacher-training program to 23 undergraduate students at two Canadian universities. The training consisted of 1.5 h per week of workshops, group discussions, workbook exercises, and homework assignments. A pre- to post-test EI measure revealed that the experimental group had a significantly positive change in the score, while the control group did not. Gilar-Corbi et al. [122] indicated a notable enhancement in the EI of students in an experimental group compared to a control group. Gilar-Corbi et al. [32] delivered a 30 h training course to 54 senior managers in a private company. They employed a pre- and post-test design with a control group. These findings indicate that EI ability was enhanced after training.

However, other studies have demonstrated no statistically significant difference between pre- and post-test EI scores [33,34]. Nevertheless, these studies have identified that the absence of a statistically significant difference between pre-test and post-test scores could be attributed to various factors, such as measurement issues of not applying 360-degree feedback, challenges associated with the Bar-On model, intervention effectiveness considering training best practices for EI, external factors, such as social and organizational support, and small sample size [33,34]. As our study sought to mitigate these concerns by adopting the ability model and incorporating social and organizational support as a moderator, including both self- and supervisor evaluation samples, with a sizable sample of 120 participants, we assumed that these factors might not apply to our study. Therefore, considering the abovementioned empirical evidence and rationale, we developed our first hypothesis as follows.

**Hypothesis 1 (H1):**  *EI training has a positive impact on learning.*

#### 2.2.2. Learning on Training Transfer

The KSAs acquired during training are key antecedents of training transfer. Based on Baldwin and Ford’s [28] first model of training effectiveness, learning and retention are considered the determinants of generalization and maintenance of training materials.

Therefore, investigating the effect of learning on transfer is crucial in an organizational context.

Several empirical studies, although not specific to EI training, have found that learning has a positive effect on training transfers. Liebermann and Hoffmann’s [123] comprehensive study demonstrated that learning affects training transfer. Velada et al. [54] found that trainees were more likely to perceive a successful transfer to the workplace if they retained the training content. Homklin et al. [26] found that learning has a positive effect on transfer. 

Based on the theoretical and empirical evidence, we developed our second hypothesis as follows.

**Hypothesis 2 (H2):**  *Learning from EI training positively impacts training transfer.*

#### 2.2.3. Mediation of Learning

In the literature, some studies reported a positive impact of EI training on training outcomes, including learning skills, abilities, and knowledge [3,116,117,118,119,120,121]. Regarding the effect of learning on training transfer, Baldwin and Ford’s [28] model explains that learning and retention are considered the determinants of training transfer (generalization and maintenance). Moreover, some empirical studies, which are not EI-specific, have revealed a positive relationship between learning and training transfer. For instance, Liebermann and Hoffmann demonstrated that learning influences training transfer, while Velada et al. [54] found that learning obtained from training is a prominent antecedent of training transfer. Moreover, according to Kirkpatrick [29], trainees need new KSAs to change their behavior in the workplace.

Building upon this evidence, we developed hypotheses to test the impacts of EI training on learning (H1) and learning from EI training on transfer (H2), as discussed in Section 2.2.1 and Section 2.2.2. Additionally, leveraging these insights, we propose mediation hypotheses to explore the indirect effect of learning on training transfer through EI training. Therefore, our third hypothesis is as follows.

**Hypothesis 3 (H3):**  *Learning positively mediates the relationship between training and training transfer.*

#### 2.2.4. Emotional Intelligence Training on Training Transfer

Miao et al. [47] suggested that EI is a crucial factor in predicting OCB because it may inspire employees to engage in OCB by enabling them to recognize and understand their own and others’ emotions. Moreover, Abraham [49] emphasized that EI performs a critical role in fostering empathic altruistic behaviors in the workplace, as well as in establishing and maintaining good social relationships. It makes employees more likely to provide empathic support and assistance to their colleagues who need help and support at work [9]. Moreover, the EI of individuals is also closely related to their positive mood [124]. Spector and Fox [48] stressed that employees in a positive mood display positive behaviors towards personal and organizational performance. In addition, Carmeli and Josman [9] found that highly emotionally intelligent individuals comprehend organizational norms and rules and are more sensitive to positive informal behaviors in the workplace.

Prior studies [9,46,49,125,126,127] consistently showed the positive effect of EI on OCB. Additionally, Permatasari et al. [128] and Goudarzian et al. [129] found that EI training can enhance OCB, supported by studies on elementary teachers. Based on the above arguments, we observed that the effect of EI training on OCB has been found in various organizational settings and conditions. We anticipate that EI training will positively affect OCB.

We can also expect an effect of EI training on CWB. EI reduces CWB by controlling negative emotions, leveraging positive emotions [130,131], and fostering positive behaviors [132,133]. This enables constructive responses to work challenges and maintains positive conduct, even in difficult situations [50]. In addition, the ability to regulate emotions plays a crucial role in establishing and sustaining positive social relationships, which are important for employee well-being [134]. This may help employees deal with stressful situations more effectively, avoid exhibiting CWB [134], and refrain from behaviors harmful to organizations [135].

Some studies found a negative correlation between EI and CWB [49,50,127,134,136]. Eniola [137] found that EI training reduced aggressive behavior in the experimental group, whereas Pozo-Rico et al. [138] observed that EI training positively affected the experimental group in preventing the negative behaviors of teachers. Considering this rationale and the empirical evidence, we expect that EI training will positively influence CWB reduction.

All studies related to the effect of EI training on OCB and CWB have investigated the overall effect, including the learning-mediated path. Since we already hypothesized the learning-mediated effect separately (H3), we needed to justify the direct effects, excluding the learning-mediated path, which has been considered to occur through improving motivation to transfer. More specifically, training is expected to affect self-efficacy, motivation to transfer, and in turn training transfer [139,140,141,142].

Therefore, EI training could help increase training transfer, even without the mediation of learning. Therefore, we develop the fourth hypothesis as follows.

**Hypothesis 4 (H4):**  *EI training has a direct, positive impact on training transfer.*

#### 2.2.5. Moderation of Social and Organizational Support

Based on SET and OST, we can presume that if there is a high level of social and organizational support readily available, employees try their best to improve organizational commitment, job engagement, performance, and behaviors [65,114,115] because high levels of social and organizational support shape employees’ confidence, attitudes, self-esteem, and overall satisfaction [65,104,143] which fosters a mutually beneficial relationship between employees 

Therefore, we can assume that when social and organizational support is at a higher level, trainees obtain newer KSAs from the training by setting new learning goals. This means that when social and organizational support is higher, the effect of EI training on learning is stronger. Based on this rationale, we develop the fifth hypothesis.

**Hypothesis 5 (H5):**  *Social and organizational support moderates the effect of EI training on learning, such that when social and organizational support is higher, the effect of EI training on learning is stronger.*

Our sixth hypothesis regards the moderating effect of social organizational support on the relationship between learning and transfers. Based on the discussion above, a higher level of social and organizational support motivates a higher level of commitment, dedication, behavior, and performance [104,143,144], and we can expect that after acquiring new KSAs from the training, they will be more motivated to transfer (behavior change) what they have learned in the training to the workplace when a higher level of social and organizational support is available. Moreover, based on the findings of Richman-Hirsch [61], Homklin et al. [26] assumed that trainees receiving strong social and organizational support would exhibit more behavioral change because of the training. Based on these arguments, we developed the sixth hypothesis.

**Hypothesis 6 (H6):**  *Social and organizational support moderates the effect of learning on training transfer, such that when social and organizational support is higher, the effect of learning on training transfer is stronger.*

Following SET, OST, and empirical evidence, when employees perceive a high level of social and organizational support, they are more motivated to apply newly obtained KSAs to the job compared with those perceiving a low level of support [145,146,147,148] because a high level of social and organizational support shapes employees’ confidence, attitudes, self-esteem, commitment, and overall satisfaction [65,104,143]. Moreover, when higher levels of social and organizational support are available, employees reciprocate by engaging in the extra role of behavior [144]. Thus, we expect that when employees perceive a higher level of social and organizational support in the organization, they will engage in OCB and prevent CWB. Moreover, Richman-Hirsch [61] emphasized that the effectiveness of post-training interventions on training transfer is moderated by perceptions of the work environment. Based on these arguments, we propose the following seventh hypothesis:

**Hypothesis 7 (H7):**  *Social and organizational support moderates the effect of EI training on training transfer, such that when social and organizational support is higher, the effect of EI training on training transfer is stronger.*

### 2.3. Conceptual Framework 

**Figure 1:**
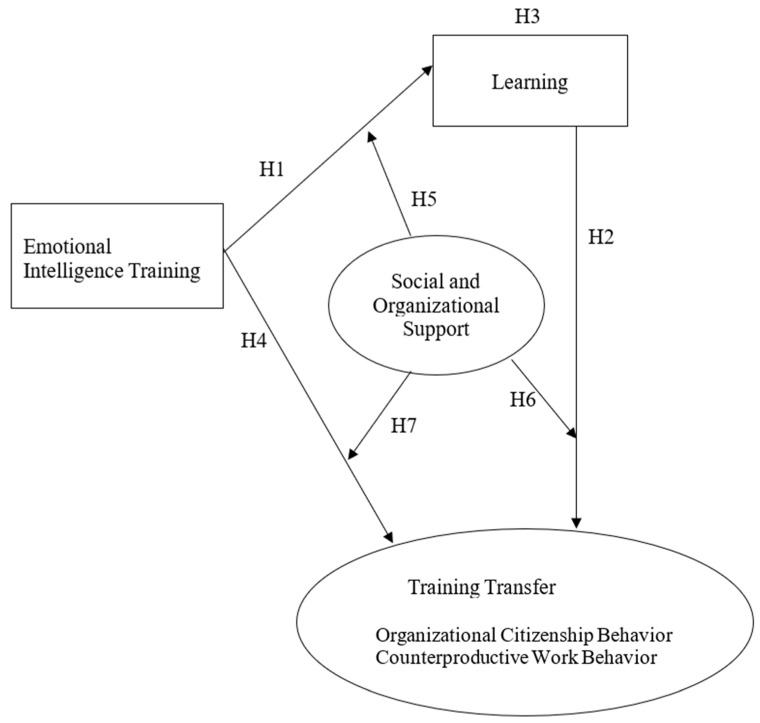
Conceptual Framework.

The conceptual framework and hypotheses are illustrated in Figure 1. 

## 3. Materials and Methods

This section is explained in several subsections. A concise and precise description of the organizational context, participants, procedures, measurements, and analysis strategy is provided.

### 3.1. Organizational Context and Training Contents

A comprehensive survey was administered to government officials in the Western Provincial Council (WPC), which is a prominent provincial administrative body in Sri Lanka with a distinguished status owing to its pivotal role in driving Sri Lanka’s socioeconomic development. In addition, the WPC functions within the framework of Sri Lanka’s decentralized governance structure, addressing regional issues and advancing the welfare of its populace, accommodating approximately 90,000 government officials in 1466 provincial council institutions that encompass a diverse array of sectors.

The Personnel and Training Division of the WPC is responsible for orchestrating and conducting training programs both domestically and internationally for government officials in the WPC and its affiliated institutions. In adherence to the guidelines delineated in the Sri Lankan Public Administration Circular [149], a mandatory requirement stipulates that all government officers must complete a minimum of 12 h of training annually. Consequently, in 2019, the WPC conducted approximately 175 training programs, which were specifically designed to cater to the developmental needs of approximately 10,000 officials, incurring an expenditure of approximately USD 100,000. Therefore, we evaluated the training provided to WPC officials. We opted for EI training from the WPC’s annual programs since it is pivotal in equipping individuals with the skills needed for management, which is imperative for improving individual and organizational performance.

### 3.2. Participants and Procedures

From the entire application pool comprising 378 applications, we adopted a randomized controlled trial approach to randomly select 176 officials who applied for the EI training program. This selection was based on the capacity limitations of the training program. Subsequently, these officials were randomly assigned to either the experimental or the control group, with each group consisting of 88 participants. Subsequently, a subject expert in the field of EI conducted a three-day course (8 h a day) covering theoretical and practical aspects of EI to enhance organizational outcomes only for treatment group officials at the WPC, Sri Lanka. 

Moreover, before the data collection, this study secured requisite ethical clearance through approval from the Research Ethics Review Board (HR-LPES-000837) of Hiroshima University in Japan, ensuring strict adherence to established ethical guidelines and protocols. Moreover, before the commencement of data collection, all participants provided informed consent for participation in our research, and their responses were kept confidential.

Officials from both groups were requested at different times to complete online Google Forms questionnaires utilizing five-point and seven-point Likert scale formats as well as a self-reported EI scale developed by Schutte [150]. To ensure linguistic congruence, all questionnaires were translated into the Sinhala language by a government-authorized translator in the WPC. A back-translation process was employed to ensure accuracy and alignment with the original version. However, since the government officials of Sri Lanka possess adequate English language proficiency, all questions were presented in both Sinhala and English languages in each Google Forms questionnaire to improve their understanding of the given questions. Due to the utilization of a longitudinal research design, all data were periodically obtained before, immediately after the training, and 1 month and 3 months after the training.

Before training (Time (0)), we utilized the Schutte Self-Report Emotional Intelligence (SSREI) scale to measure the ability level related to EI of all the officials in both treatment and control groups to assess the learning outcome. As we mentioned above, we measured the training transfer outcome using behavior change in OCB and CWB. Assessing behavior change of both participants and supervisors has been recommended by Chiaburu et al. [151] for future research which concentrates on acquiring training transfer ratings to achieve a comprehensive measurement of training transfer. They found that supervisors evaluated employee skill transfer more accurately when newly transferred skills were observable. Their study highlighted the potentially critical role of supervisors as the most reliable judges and sources of ratings for transfers, although Carpenter et al. [152] showed that CWB was not observable by supervisors and coworkers. In terms of OCB, Allen et al. [153] confirmed that the measurement results did not differ between self- and supervisor evaluation samples. Therefore, a more reliable source should not be predetermined. As Haccoun and Hamtiaux [154] emphasized, obtaining training transfer ratings from participants and their supervisors helps determine whether respondents’ transfer estimates are accurate. Therefore, to check the robustness of participants’ responses in both treatment and control groups, before the training, we gave a questionnaire regarding OCB and CWB to the participants and their respective immediate supervisors to measure the initial levels of OCB and CWB. Subsequently, the supervisors provided their assessments of the OCB and CWB levels exhibited by their subordinates who took part in this study. In addition, participants self-assessed their levels of OCB and CWB before the training program (Time (0)). Furthermore, social and organizational support was assessed before the training. Moreover, participants provided demographic information, including name, age, sex, place of employment, level of education, and years of experience in the public sector, as control variables. Afterwards, the employees in the treatment group underwent a three-day EI training and were explicitly instructed not to share the acquired knowledge with others to prevent any potential spillover effects. Immediately after the training (Time (1)), the same self-reported EI scale was administered to measure the ability level after training.

Baldwin [28] and Noe and Schmitt [155] suggested that the immediate period after training is important for training transfer. Axtell et al. [156] indicated that most training transfers may occur in the first month. Also, they have recommended that behavior data should be collected one month after the training because it is expected to predict the behavior for the long term.

Therefore, 1 month after training (Time (2)), we again gathered self-assessments of OCB and CWB levels from participants utilizing the same questionnaires of OCB and CWB. However, at this time we did not provide OCB- and CWB-related questionnaires to the participants’ supervisors to obtain their assessment of their subordinates’ levels of OCB and CWB because of their busy work schedules and we received a low response rate before the training program. Following Kirkpatrick’s [157] recommendations, and to allow sufficient time for trainees to apply their KSAs, 3 months after the training (Time (3)), we again administered the same OCB and CWB questionnaires to both participants and their supervisors. The summary of variables measured at each time point as outlined in Table 1 is as follows.

### 3.3. Measures

We utilized 126 measurement items that have been previously validated in the literature. All measurement items are listed in Appendix A.

#### 3.3.1. Learning

In our study, we considered change in KSAs, or ability, as learning. Therefore, we utilized the change in the ability levels from the ability test result from “before” to “just after” the training to measure the learning. Therefore, the levels of ability of EI of each participant were measured using a five-point Likert scale, a self-reported EI scale developed by Schutte et al. [150] before and just after the training. The SSREI is one of the ability models consisting of prominent self-report measures used to assess EI abilities, comprising a 33-item self-report ability-based EI scale developed based on Salovey and Mayer’s model [68] of EI. The SSREI scale evaluates an individual’s level of self-awareness in comprehending and effectively handling their own emotions and the emotions of others and measures how it impacts their ability to solve problems [150,158]. Sample questions included “I like to share my emotions with others” and “When I am in a positive mood, solving problems is easy for me”. Respondents rated their agreement with each statement on a five-point Likert scale (1 = strongly disagree, 2 = disagree, 3 = neutral, 4 = agree, and 5 = strongly agree). Schutte et al. [150] found high internal consistency (Cronbach’s alpha, α = 0.90) and test–retest reliability of α = 0.78 over a two-week interval for a smaller subgroup within the sample.

#### 3.3.2. Training Transfer/Behavioral Change

The level of behavior at different time frames was assessed by measuring the levels of OCB and CWB using both self-evaluation and supervisor evaluation samples. Twenty-four OCB measures were adopted for both self- and supervisor evaluation samples from the study of Podsakoff et al. [159]. This scale assesses voluntary behaviors beyond job requirements that enhance organizational effectiveness, including five factors, namely, altruism (helping others selflessly), courtesy (politeness and respect), civic virtue (active participation in organizational activities), conscientiousness (diligent and reliable work), and sportsmanship (positive attitude under pressure). The items in the self-OCB evaluation included “I try to avoid creating problems for coworkers” and “I do not abuse the rights of others”. The items in the supervisors’ OCB evaluation include “This officer tries to avoid creating problems for coworkers” and “This officer does not abuse the rights of others”. Respondents rated their level of agreement with each statement using a seven-point Likert scale (1: strongly disagree, 2: disagree, 3: somewhat disagree, 4: neutral, 5: somewhat agree, 6: agree, 7: strongly agree). Podsakoff et al. [159] reported that the internal consistency reliability of the five subscales was above 0.8, except for items of civic virtue, which had a reliability coefficient of α = 0.7.

The level of CWB was measured for both self- and supervisor evaluation samples by utilizing 45 items, including the CWB scale by Spector et al. [130], a widely used measurement tool to assess CWB in the workplace. The scale comprises several items designed to capture various dimensions of CWB, such as theft, sabotage, absenteeism, lateness, gossiping, or other forms of misconduct. Sample items for self-evaluation include “I purposely did my work incorrectly” and “I verbally abused someone at work”. Sample items for supervisor evaluation sample include “This officer purposely did their work incorrectly” and “This officer has verbally abused someone at work”. Respondents rated the extent to which they had engaged in or witnessed such behaviors on a five-point Likert scale (1: never, 2: once or twice, 3: once or twice per month, 4: once or twice per week, and 5: every day). Spector et al. [130] found a substantial reliability level with Cronbach’s alpha of 0.9 for their measurements.

#### 3.3.3. Organizational and Social Support

A total of 25 social and organizational support measures were derived from Holton et al. [160] and Kupritz [161] to assess the support level in terms of the training transfer climate. Social support encompasses both supervisor support and coworker support. The sample items for supervisor support are “My supervisor sets criteria for applying new KSA to my job” and “My supervisor discusses how to apply KSA to the job situation”. The sample items for coworker support include “My coworker cares about my applying new knowledge and skills on the job” and “My coworker frequently shares work-related information/knowledge with me”.

Items that provide organizational support include “My organization has a strategy plan and interest in the personal and professional development of employees” and “My organization has an inefficient and inflexible workspace for teaching knowledge and skills from training to other employees”. Respondents rated the extent to which they agreed with each statement on a five-point Likert scale of both social and organizational support (1: strongly disagree to 5: strongly agree). Holton et al. [160] indicated Cronbach’s alpha exceeding 0.7 for the social support scale. Additionally, a Cronbach’s alpha above 0.6 was reported for the organizational support scale [26].

### 3.4. Analysis Strategy

An experimental research design was used to compare the training outcomes between the treatment and control groups using RCT methodology. The hypothesized model was evaluated using SPSS software (version 28.0). As a preliminary analysis, the two groups were compared using *t*-tests and chi-square tests for demographic characteristics and training outcome variables (learning and training transfer) before the EI training.

We performed mediation analysis using Hayes’ Process Macros in SPSS to test the hypotheses for both the direct and mediation paths. Hierarchical regression analyses were performed to examine the moderating effects of social and organizational support on the relationship between training and its outcomes. Additionally, to explore the significant variations in OCB and CWB over different time intervals, a post hoc analysis was conducted after analysis of variance (ANOVA). To analyze the training transfer, we analyze the change in OCB and CWB levels from the period “before” to “1 month after” the training (Transfer 1) only in the self-evaluation sample since we did not collect the data from the supervisors 1 month after the training. Further, we analyzed the change in OCB and CWB levels from the period “before” to “3 months after” the training (Transfer 2) in both self-evaluation and supervisor evaluation samples.

## 4. Results

### 4.1. Descriptive Statistics of the Demographic Traits, Balance Check, and Correlation Matrix

This section presents the descriptive statistics based on the demographic characteristics, results of the balance check, and details on the correlation matrix including the demographic variables and other variables that we utilized in our main analysis in both samples. The total number of employees in both treatment and control groups is 176, with each group comprising 88 participants. We achieved a 100% response rate from the participants for all distributed questionnaires through effective awareness and guidance. Though we sent the OCB and CWB questionnaire to 19 supervisors to obtain their observation of the OCB and CWB levels exhibited by 176 subordinates, the response rate was 68% (we received only 119 responses from 14 supervisors) before training and 44% (we received only 78 responses from 9 supervisors) 3 months after training. Therefore, a non-response bias analysis (Table S2) was conducted for the supervisor evaluation sample; as a result, we found that there was no significant difference between those who responded and those who did not. The responses from the self-evaluation sample, totaling 176, and the supervisor evaluation sample, comprising 78 responses three months after the training, were analyzed separately.

The 176 participants of the self-evaluation sample comprised 88% women and 12% men. In terms of age, 4% were 20–30 years old, 64% were 31–40 years old, 25% were 41–50 years old, and 7% were 51–60 years old. Among officials, 38% had a high school education, 56% held a bachelor’s degree, and 6% held a master’s degree. In terms of experience in the government sector, 56% of government officials had 0–10 years, 29% had 11–20 years, and 15% had 21–30 years. In terms of the supervisor evaluation sample, it was observed that 88% were women and 12% were men. According to age, 2% of respondents were 20–30 years old, 64% were 31–40 years old, 27% were 41–50 years old, and 7% were 51–60 years old. In this sample, 37% held a high school education, 55% held a bachelor’s degree, and 8% held a master’s degree. In terms of government sector experience, 53% had between 0 and 10 years, 30% had 11 to 20 years, and 17% had 21 to 30 years. Table 2 outlines the demographic characteristics of the self-evaluation and supervisor evaluation samples.

Table 3 presents a summary of the means, standard deviations (SDs), and correlation of all the variables including the demographic variables and other variables which we have utilized in our main analysis in both the self-evaluation and supervisor evaluation samples. This study’s objective is to identify the effect of the training on the change in ability as learning and that of behavior as training transfer. Therefore, we analyzed the change in the ability levels through the ability test result from the period “before” to “just after” the training and changes in OCB and CWB as Transfer 1 and Transfer 2. All the variables that we utilized in our main analysis and the demographic characteristics of the self-evaluation and supervisor evaluation samples are displayed in Table 3. The correlation matrix indicated a significant relationship between learning and changes in OCB and CWB. However, demographic characteristics did not show significant correlations with other variables.

Furthermore, the likelihood ratio chi-square test was employed to assess the demographic data distribution between the treatment and control groups for both the self- and supervisor evaluation samples, for sex (self-evaluation sample: χ^2^ = 0.054, *p =* 0.816, supervisor evaluation sample: χ^2^ = 2.121, *p* = 0.145), workplace (self-evaluation sample: χ^2^ = 0.024, *p* = 0.878, supervisor evaluation sample: χ^2^ = 0.203, *p* = 0.652), and educational qualification (self-evaluation sample: χ^2^ = 1.384, *p* = 0.501, supervisor evaluation sample: χ^2^ = 0.424, *p* = 0.809). Independent *t*-tests were conducted to compare the age and years of public service between the groups. The results revealed no significant differences in age (self-evaluation sample: mean difference (MD) = 0.693, *p* = 0.446, supervisor evaluation sample: MD = 1.090, *p* = 0.340) and years in public service (self-evaluation sample: MD = 0.648, *p =* 0.544, supervisor evaluation sample: MD = −0.035, *p* = 0.978) between the groups. These findings indicate that our random assignment was successful as there were no noticeable differences in demographic characteristics between the two groups (Table S3).

### 4.2. Results of Reliability and Validity Test

To check the reliability and validity of the measures, we performed a reliability and validity test using the SPSS software (version 28.0) with measurements for self-evaluation and supervisor evaluation samples carried out separately. The reliability test revealed that Cronbach’s alpha values of each variable exceeded the standard threshold of 0.7 [162], and it can be concluded that all the variables exhibited strong reliability (Table 4). We assessed the validity of the measurement through convergent validity and discriminant validity tests. To evaluate convergent validity, we conducted factor analysis in SPSS for all the constructs. Subsequently, average variance extracted (AVE) values were calculated to assess convergent validity. The analysis revealed an AVE score of all the measurements exceeding the minimum criterion of 0.5 [163]. The results confirm the convergent validity of the variables (Table 4). 

To verify discriminant validity, we computed the square root of the AVE for each variable. When the square root of a variable’s AVE exceeds the correlation coefficients with all other variables, it meets the criteria for discriminant validity [163]. Since the square roots of all variables surpassed the correlation coefficients, our measurements met the requirements for demonstrating discriminant validity (Table 5).

### 4.3. Results of the Preliminary Analysis

Before training (Time (0)), the independent samples *t*-test revealed no statistically significant differences in the outcome variables between the treatment and control groups. The analysis revealed no significant difference between the two groups in terms of ability (MD = 1.545, *p =* 0.485) (Table 6). In addition, no significant differences were found between the two groups in the self-evaluation samples for OCB (MD = 1.120, *p* = 0.548) and CWB (MD = −0.750, *p* = 0.750). Similarly, no significant differences were observed in the supervisor evaluation sample for OCB (MD = 0.651, *p* = 0.806) and CWB (MD = −5.890, *p* = 0.339) (Table 7). These findings suggest that the random assignment of participants was successful in terms of the outcome variables.

An independent samples *t*-test was conducted to compare the EI ability test result between the periods “before” and “just after” the training (Time (1)) between the treatment and control groups. Statistically significant differences in the mean values (MD = 16.011, 95% confidence interval: 12.745–19.278) were observed between the two groups (Table 6). There was a significant difference in the mean scores between the treatment group (M = 16.09, SD = 13.373) and the control group (M = 0.08, SD = 7.889) (Table 6).

To examine OCB and CWB changes between the two groups at Time (2) and Time (3), independent samples *t*-tests were conducted based on self-evaluations and supervisors’ evaluations (Table 7). Significant disparities were found between the treatment and control groups in both self- and supervisor evaluation samples, specifically regarding Transfer 1 and Transfer 2. We found that OCB had higher scores in the self-evaluation (Transfer 1: MD = 12.557, *p* = 0.000; Transfer 2: MD = 10.955, *p* = 0.000) and supervisor evaluation samples (Transfer 2: MD = 16.429, *p* = 0.000). We also found the same results for change in CWB (self-evaluation sample: Transfer 1: MD = −8.614, *p* = 0.000; Transfer 2: MD = −7.136, *p* = 0.000; supervisor evaluation sample: Transfer 2: MD = −14.033, *p* = 0.011).

**Figure 2:**
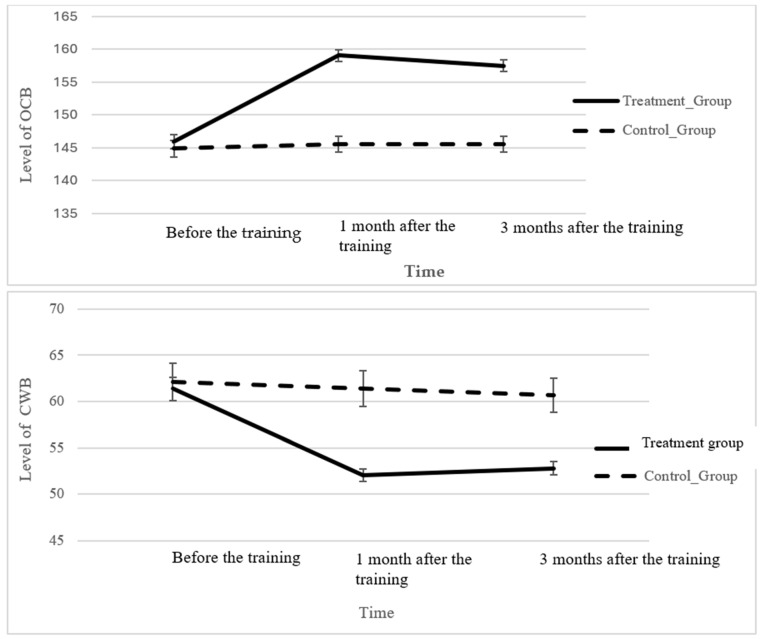
Results of Multiple Group Comparisons: OCB and CWB at Different Times.

To explore significant variations in OCB and CWB levels over different time intervals, a post hoc analysis was conducted for the self-evaluation sample after ANOVA. This analysis aimed to compare the group means and identify the specific time points at which significant differences were observed. The results revealed a substantial divergence in OCB and CWB levels between the treatment and control groups 1 month and 3 months after the training (Table 8). However, no significant differences were found in OCB and CWB levels 1 month and 3 months after the training, suggesting consistent levels of OCB and CWB for 1 month and 3 months after training (Figure 2).

### 4.4. Testing the Hypotheses

For hypothesis testing, we employed the change in ability as a measure of learning. This was assessed by comparing scores of ability tests administered before and immediately after the training. Also, to measure the OCB and CWB change, we utilized OCB and CWB change as Transfer 1 and Transfer 2 for the main analysis of each of the self- and supervisor samples. To test the effect of training-on-training transfer on the mediation of learning, we used the Process Macro developed by Hayes et al. [164] in SPSS 28. Table 9 shows the regression outcomes of the mediation model of the self-evaluation sample of OCB and CWB in Transfers 1 and 2 and the supervisor evaluation samples of Transfer 2. 

The first model of each OCB and CWB analysis of the self-evaluation and supervisor evaluation samples assessed the effects of EI training on learning. Significant positive results emerged (β = 15.951, *p* = 0.000), with R^2^ = 0.361 in the analyses of self-evaluation. It showed a positive significant effect of EI training on learning (β = 14.569, *p* = 0.000, R^2^ = 0.363) in the case of the supervisor evaluation sample. Consequently, the results confirm that EI training has a positive impact on learning. Hence, H1 is supported. 

H2 posits that learning would positively affect training transfer. As previously mentioned, we anticipated that learning would exert a positive impact on OCB and a negative impact on CWB. However, the findings presented in Table 9 indicate that EI training has a positive influence on OCB only in the self-evaluation sample and not in the supervisor evaluation sample (self-evaluation sample, Transfer 1: β = 0.139, *p* = 0.000, R^2^ = 0.469, self-evaluation sample, Transfer 2: β = 120, *p* = 0.000, R^2^ = 0.426, and supervisor evaluation sample, Transfer 2: β = −0.212, *p* = 0.138, R^2^ = 0.348). Additionally, EI training exhibits a negative impact on CWB only in the self-evaluation and not in the supervisor evaluation sample (self-evaluation sample, Transfer 1: β = −0.234, *p* = 0.000, R^2^ = 0.346, Transfer 2: β = −0.200, *p* = 0.052, R^2^ = 0.264, and supervisor evaluation sample, Transfer 2: β = 0.407, *p* = 0.109, R^2^ = 0.203). These results confirm that learning has a positive impact on training transfer only in self-evaluation, partially supporting H2.

H3 concerns the mediating effect of learning between training and transfer. The result indicated partial mediation of the learning between training and transfer in the OCB change and CWB change as Transfer 1 and Transfer 2 of the self-evaluation sample (Transfer 1 OCB: β = 2.215, LLCI = 0.948, ULCI = 4.015, Transfer 2 OCB: β = 1.917, LLCI = 0.699, ULCI = 3.596, Transfer 1 CWB: β = −3.734, LLCI = −5.636, ULCI = −1.579, Transfer 2 CWB: β = −3.195, LLCI = −4.997, ULCI = −1.164). Furthermore, we analyzed the mediation effect for the supervisor evaluation sample, however, we could not see any mediation effect of learning on the relationship between training and OCB and CWB change as Transfer 2 (OCB change as Transfer 2: β = 3.097, LLCI = −7.308, ULCI = 1.537, CWB change as Transfer 2 β = 5.853, LLCI = −0.952, ULCI = 13.887). Therefore, H3 was partially supported (Table 10).

H4 posits that EI training exerts a positive effect on training transfer directly, without the mediation of learning. Since we gauged training transfer through changes in levels of OCB and CWB, we anticipated that EI training would yield a positive effect on OCB change and a negative effect on CWB change. As anticipated, Table 9 reveals that for Transfer 1 (β = 10.384, *p* = 0.000, R^2^ = 0.469) and Transfer 2 (β = 9.111, *p* = 0.000, R^2^ = 0.426) in the self-evaluation sample and Transfer 2 (β = 10.296, *p* = 0.000, R^2^ = 0.348) in the supervisor evaluation sample, EI training positively affects the OCB. Additionally, EI training negatively affects the CWB change (self-evaluation sample, Transfer 1: β = −4.773, *p* = 0.000, R^2^ = 0.346, Transfer 2: β = −3.898, *p* = 0.006, R^2^ = 0.264 and supervisor evaluation sample Transfer 2: β = −20.413, *p* = 0.002, R^2^ = 0.203). This demonstrates that EI training has the expected impact on training transfer. This finding provides substantial empirical support for H4. 

**Figure 3:**
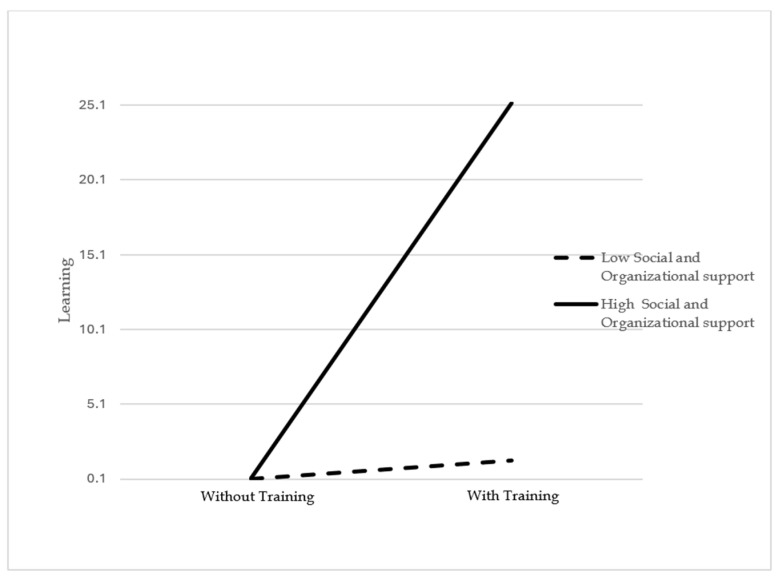
Moderation Effect of Social and Organizational Support on the Relationship between Training and Learning in Self-Evaluation Sample.

Furthermore, hierarchical regression analysis was employed to examine the moderation hypotheses. The results of the moderation analysis presented in Table 11 indicated a significant strengthening moderation of social and organizational support on the relationship between EI training and learning in the self-evaluation sample (β = 0.413, *p* = 0.000). Figure 3 illustrates the interaction between social and organizational support and EI training on learning in the self-evaluation sample. Therefore, H5 was supported. Since we did not obtain feedback from the supervisors regarding the learning level of participants, we did not analyze the moderation effect of social and organizational support on the effect of EI training on learning in the supervisor evaluation sample. 

H6 is that social and organizational support moderates the relationship between learning and training transfer. We conducted separate analyses for the self-evaluation and supervisor evaluation samples concerning Transfer 1 and Transfer 2. Our observations indicate the absence of moderation in each of these evaluations (self-evaluation sample OCB change as Transfer 1: β = −0.330, *p* = 0.873; self-evaluation sample OCB change as Transfer 2: β = −0.079, *p* = 0.458, self-evaluation sample CWB change as Transfer 1: β = −0.155, *p* = 0.134; self-evaluation sample CWB change as Transfer 2: β = −0.124, *p* = 0.249, supervisor evaluation sample OCB change as Transfer 2: β = −0.167, *p* = 0.247; supervisor evaluation sample CWB change as Transfer 2: β = 0.067, *p* = 0.648). Therefore, H6 was not supported (Table 12).

**Figure 4:**
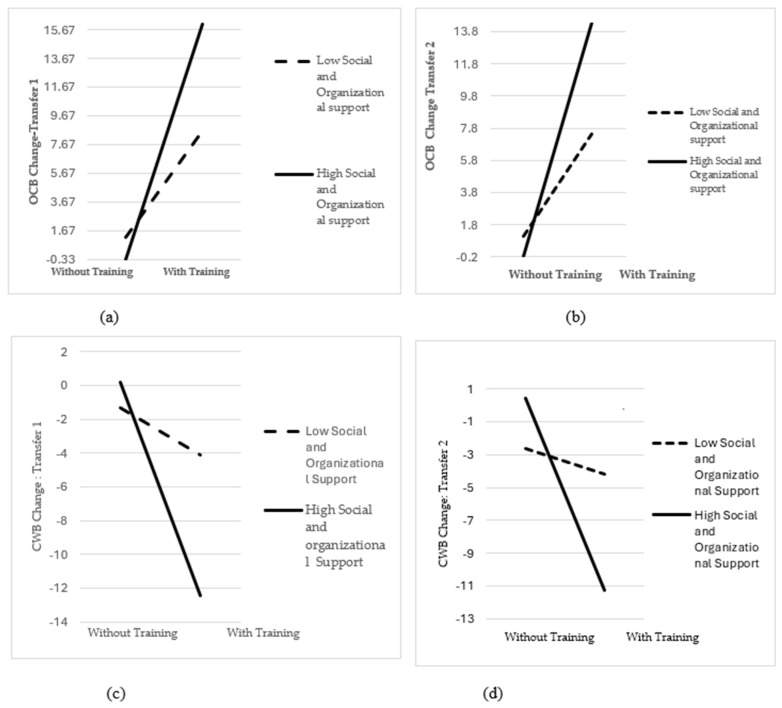
Moderation Analyses of the Moderation Effect of Social and Organizational Support on the Relationship between Training and (**a**,**b**) OCB and (**c**,**d**) CWB in Self-Evaluation Sample. (**a**) OCB change between before the training to 1 month to after training (Transfer 1); (**b**) OCB change from before the training to 3 months after training (Transfer 2); (**c**) CWB change from before the training to 1 month after training (Transfer 1); (**d**) CWB change from before the training to 3 months after training (Transfer 2).

Furthermore, moderation analyses were performed on the direct relationship between EI training and OCB as well as CWB change in the self- and supervisor evaluations according to Transfer 1 and Transfer 2. The findings of self-evaluation indicated that social and organizational support had a strengthening moderation on the relationship between EI training and changes in OCB and CWB as Transfer 1 and Transfer 2 (OCB change as Transfer 1: β = 0.215 *p* = 0.000, OCB change as Transfer 2: β = 0.202, *p* = 0.000, CWB change as Transfer 1: β = −0.237, *p* = 0.001, CWB change as Transfer 2: β = −0.261, *p* = 0.000) (Figure 4). Moreover, we could not see any moderation effect of social and organizational support on the relationship between training and OCB and CWB change in the supervisor evaluation sample (Table S4) for both Transfer 2 in OCB and CWB (OCB change as Transfer 2: β = −0.018, *p* = 0.873; CWB change as Transfer 2: β = −0.042, *p* = 0.738). Therefore, H7 was partially supported (Table 13).

## 5. Discussion

### 5.1. Discussion

This study investigates the effectiveness of EI training on training transfer (OCB and CWB change), partially mediated by learning, with the moderation of social and organizational support, to address the existing gap in the literature. As shown in the previous section, regarding the partially mediated main relationship, H1 (EI training–learning) and H4 (EI training–transfer) were supported while H2 (learning–transfer) and H3 (EI training–learning–transfer) were partially supported. Conversely, H5 (EI training–learning) was supported, H7 (EI training–transfer) was partially supported, and H6 (on learning–transfer) was not supported. These results are discussed below.

Regarding the main relationship, based on our findings, EI training has a positive effect on learning, aligning with some prior studies (H1) [30,31,32], although other studies have reported that EI training is ineffective in terms of learning EI [33,34]. Therefore, our study provides empirical evidence supporting the notion that adults can be trained through EI training to acquire corresponding KSAs. Moreover, our study shows that EI training has a positive effect on training transfer also. These results underscore the added value of EI training in facilitating both the acquisition of KSAs and their application in employees’ jobs. In addition, by paying attention to key EI competencies such as self-awareness, social awareness, self-management, and relationship management [79], EI training equips people with the necessary tools to build up interpersonal relationships, manage emotions, and adapt to changing circumstances. Further, in our study, we observed that OCB and CWB can be cultivated, demonstrating the potential for EI training to contribute significantly to organizational success. Thus, our study highlights the considerable added value of EI training as a strategic investment in human resource development, with implications and potential benefits for both individuals and organizations.

Next, although we expected learning to have a positive effect on transfer/behavior changes from both the analysis of self-evaluation and supervisor evaluation, our observations indicate that the positive effect of learning on transfer is evident solely within the self-evaluation sample and not within the supervisor evaluation sample (H2). The positive effect of learning on transfer in the self-evaluation sample is confirmed by previous studies [26,28,41,123]. However, the supervisor evaluation sample did not demonstrate a positive effect on learning or transfer. The H1 and H2 results revealed a partial mediation of learning between training and transfer in OCB and CWB as Transfer 1 and Transfer 2 of the self-evaluation sample. Furthermore, due to the findings on H2, we did not observe any mediation effect in the supervisor evaluation sample. The different results for the two samples are elaborated on in more detail at the end of this section.

Moreover, our results indicate that EI training has a positive effect on training transfer (H4); in particular, EI training has a positive effect on OCB [9,46,49,125,126,127] and a negative effect on CWB [49,50,127,134,136] 1 month and 3 months after the EI training in both self- and supervisor evaluation samples. This validates the findings of previous studies and confirms their results regarding the direct effect of EI training on training transfer [32,165]. 

Next, let us discuss moderation. Both self- and supervisor evaluation samples demonstrated a notable strengthening impact of social and organizational support in the relationship between EI training and learning (H5). Therefore, it confirmed SET and OST in that when social and organizational support is high, trainees obtain new KSAs more so than when there is a low level of social and organizational support. 

In contrast, the relationship between learning and transfer was not moderated by social and organizational support (H6). These results aligned with the findings of Homklin et al. [26] as their study similarly indicated that the moderation effect of social (especially supervisor support) and organizational support was not present in the relationship between learning and transfer. Certain research has indicated that various types of support for training transfer outcomes might be disrupted by cultural differences within an organization [166,167]. Additionally, the presence of adverse communication content may result in counteractive buffering effects in moderating social and organizational support [168]. Consequently, we can assume that other external factors may influence social and organizational support in the relationship between learning and transfer. 

Lastly, we found inconclusive results between the self- and supervisor evaluation moderation analyses of the effect of EI training on OCB and CWB according to Transfer 1 and Transfer 2 (H7). More specifically, social and organizational support had a strengthening moderating effect on the self-evaluation sample; however, we did not observe any moderating effect of social and organizational support on the relationship between training and OCB and CWB change in the supervisor evaluation sample. We may assume that the same reasons mentioned earlier for the H6 results for both samples apply to this moderation in the supervisor evaluation sample, although we may also attribute the reason to measurement bias by the evaluator, as suggested for the H2 (and consequently H3) result.

As mentioned above, we argue for the potential sources of measurement bias for the two samples. However, we should emphasize that we obtained consistent results for some other hypotheses, which may mean that the bias did not strongly affect the results. In this regard, the argument below is not convincing enough, and thus, we need to explore this matter further.

First, before the theoretical discussion, we should pay attention to the smaller size of the supervisor evaluation sample compared to self-evaluations, which raises concerns about the reliability of the findings in the supervisor sample. This size difference may lead to statistically insignificant outcomes, thereby reducing the ability of the study to accurately detect the true effects.

Theoretical perspectives should also be considered. Empirical research has consistently indicated a notable lack of agreement between self-assessment and evaluations from external sources [169,170,171,172]. Mabe and West [173] reviewed several studies and found, on average, a low correlation between self-ratings and others’ ratings, including supervisor evaluation. Hence, these studies provide evidence that the two evaluation methods yield disparate results. 

When evaluating the reliability of self and supervisor evaluation, the extant literature emphasizes that the reliability of supervisor evaluation is more satisfactory than that of self-evaluation [174,175]. Some studies have emphasized that self-evaluations can be perceived as subjective and might not accurately reflect performance [174,176]. Other studies have shown that, as per the social desirability bias, self-assessment has a higher level of rating than supervisor evaluation, since individuals may be inclined towards perceiving themselves in an especially positive light, particularly on more desirable dimensions [177,178], while self-overestimation may boost confidence and motivation; it also raises concerns about accuracy and self-awareness. Therefore, we observed an overestimation of the self-evaluation in our study. 

Contrary to studies supporting supervisor evaluation, some argue that self-evaluation can also be deemed reliable [169,179,180]. In this line of discussion, Fox and Dinur [180] stressed that individuals are frequently in the most suitable position to accurately assess their abilities and behaviors. Jones and Nisbett [181] proposed self-perception theories, highlighting that individuals have access to a broad range of behaviors in diverse situations and over different periods. Additionally, they possess a unique ability to recognize their internal states, emotions, and dispositions and learn how they change during development. Furthermore, Harms and Crede [182] revealed that individuals with higher EI, commonly developed through training, may exhibit a better understanding and regulation of their behaviors, and potentially make self-evaluations more accurate. In addition, empirical studies have shown that individuals exhibit greater sensitivity than external observers to the situational factors influencing their behavior [180,183]. 

Additionally, observability may be another factor in the contradictory results of self- and supervisor evaluations since some changes may be more observable to the individuals themselves than to their supervisors [184]. Hence, in our study, OCB and CWB changes might not be observable to supervisors because they are voluntary behaviors. Furthermore, Kolm and Verhulst [169] emphasized that supervisors tend to evaluate their subordinates based on a global overall impression rather than on actual performance in each area. Conversely, trainees tend to differentiate levels of performance in each area.

### 5.2. Theoretical Implications

According to SET and OST, individuals with high levels of social and organizational support are more inclined to learn from and transfer training than those with low levels of social and organizational support [104,110,185]. The results of this study provide a more nuanced perspective. The findings revealed that social and organizational support moderated the effects of training on learning in both samples. However, we did not observe a moderating effect of social and organizational support on the effect of learning on training transfer. These results underscore the need for a more nuanced understanding of the factors influencing the transfer process, suggesting that contextual elements beyond social and organizational support, such as cultural differences [186] and adverse communication content [166,167,168], may play crucial roles in this process and those factors weaken the moderation of social and organizational support as we explained in Section 5.1. 

Further, evidence suggests that resource availability [57] and opportunities to apply new learning [28] greatly affect the transfer of what is learned from the training to the workplace more than other processes in training transfer since adequate resources and opportunities are essential for reinforcing learning application for effective transfer. Therefore, these findings imply that other external factors might influence social and organizational support in the relationship between learning and transfer, emphasizing the complexity of this dynamic interplay. These findings advance the discussion on SET and OST by showing the boundary conditions of their applicability. Hence, the findings contribute to redefining the existing theories and emphasizing the need for further theoretical development.

We used the EI ability model to measure learning before and after the training program. Confirmation of the EI ability model in the context of cultivating EI learning in terms of EI KSAs reinforces its theoretical validity. This implies that the model accurately captures the four essential dimensions of EI: perceiving emotions, facilitating thought using emotions, understanding emotions, and managing emotions [72,75], establishing the ability model as a dependable framework for comprehending and cultivating EI.

### 5.3. Practical Implications

This study has practical implications for enhancing the KSAs of EI and transferring them to the workplace. First, the study found that EI training is effective in improving the KSAs of EI and change in OCB and CWB, giving organizations increased confidence to invest in it, although the relationship between learning and transfer was not robust. Additionally, this research suggests that EI training has a significant potential to foster positive changes in OCB and CWB, challenging the notion that such EI training is ineffective in changing behavior and learning due to inconclusive results on the trainability of EI in the extant literature. This may offer a practical avenue for organizations and individuals to address EI and they may be trained to utilize EI training interventions and improve both learning and transfer. Moreover, it has been demonstrated that by fostering changes in behavior related to OCB and mitigating CWB, EI training has the potential to enhance extra voluntary behavior beyond regular job duties and responsibilities. Therefore, organizations should conduct EI training programs to obtain beneficial advantages and improve training effectiveness, including learning and transfer.

Moreover, the findings presented in this study highlight the crucial role played by elevated levels of social and organizational support in the better implementation of training transfer within the workplace, although such moderation was not robust, partly due to the relationship moderated by the evaluator of OCB/CWB. Therefore, to enhance training transfer effectiveness, organizations may want to cultivate social support from coworkers and supervisors as well as organizational support within their organizations. In this regard, it may be essential for organizations to improve their social and organizational support systems to take advantage of EI training for sustainable and long-term success. Specifically, organizations can exhibit social and organizational support through various practices, such as showing concern for their opinions and providing opportunities for applying KSAs to the workplace and personal and professional well-being, taking into consideration their career aspirations, recognizing and appreciating their contributions, and providing feedback [187,188].

## 6. Conclusions

This study examined the effect of EI training on training transfer, partially mediated by learning, and the relatively underexplored moderation role of social and organizational support in the main effect using an experimental and longitudinal research design. As hypothesized, the results supported the positive and significant effects of training-on-training outcomes. Moreover, social and organizational support positively moderated the effect of training on learning and training transfer in the self-evaluation sample. In addition, we did not find any significant moderating effect of social or organizational support on the effect of learning on training transfer in either sample.

## 7. Limitations and Future Research Directions

Future studies should ascertain the causal effect of learning on transfer using causal mediation analysis. This analytical approach, which was not employed in the current study, can provide valuable insights into the mediating factors influencing the relationship between learning and transfer. This study used a sample of government employees from only one organization. Therefore, the results cannot be generalized. Therefore, it would be useful to conduct future research in different organizational settings. Because this study was tailored to a specific training program, its findings might not be generalizable to other training programs. Replicating this study across diverse training contexts is necessary to ascertain the broader applicability of the results. Given that our investigation focused solely on one moderation variable, it is essential to expand its scope by exploring various mediators and moderators, as suggested by Kotsou et al. [15]. Examples include collaboration in the workplace [189] and aspects of organizational culture [167,190,191]. This broader exploration would contribute to a more comprehensive understanding of the dynamics involved in our study. Furthermore, future research could extend its focus to include other training outcomes, namely results and return on investment, which have the potential to confer competitive advantages, since our study delved into learning and training transfer only [16]. Moreover, this study evaluated the effectiveness of EI training at the individual level by focusing on individual learning and training transfer. Hence, future studies should explore team and organizational levels to assess the overall performance of organizations in terms of EI effectiveness [33]. Further, since our study points to some disparities in the self- and supervisor behavior assessment results, we suggest that future studies can further analyze the training transfer process by utilizing the self- and supervisor assessments to identify the most reliable source of observations.

## Figures and Tables

**Table 1:** The summary of variables measured at each time point.

Data Collection Time	Variable
Time (0): Before the training	Ability level, Social and organizational support, OCB level, CWB level (through both self- and supervisor assessment)
Time (1): Just after the training	Ability level
Time (2): One month after the training	OCB level and CWB level (only through self-assessment)
Time (3): Three months after the training	OCB level and CWB level (through both self- and supervisor assessment)

**Table 2:** Demographic characteristics of self-evaluation and supervisor evaluation samples.

	Self-Evaluation Sample (*n* = 176)		Supervisor Evaluation Sample (*n* = 78)	
	Frequency	Percentage	Frequency	Percentage
Sex				
Male	21	12%	51	12%
Female	155	88%	27	88%
Age				
20–30	7	4%	2	2%
31–40	113	64%	50	64%
41–50	44	25%	20	27%
51–60	12	7%	6	7%
Educational qualification				
High school	67	38%	29	37%
Bachelor’s degree	99	56%	43	55%
Master’s degree	10	6%	6	8%
Experience in the government sector				
0–10 years	99	56%	42	53%
11–20 years	51	29%	23	30%
21–30 years	26	15%	13	17%

Note: *n* = sample size.

**Table 3:** Means, Standard Deviation, and Intercorrelations of Variables of both samples.

(a) Self-Evaluation Sample (*n* = 176)														
	Minimum	Maximum	Mean	SD	1	2	3	4	5	6	7	8	9	10
1. Training	0	1	0.5	0.51										
2. Age	27	55	39.49	6.01	0.06									
3. Gender	1	2	1.12	0.032	−0.02	−0.03								
4. No. years in public service	1	30	11.71	7.04	0.05	0.71 **	−0.07							
5. Educational qualifications	1	3	2.32	0.57	−0.09	0.23 **	0.07	0.38 **						
6. Learning	−19	80	8.09	13.57	0.59 **	0.06	0.01	0.11	−0.02					
7. Social and organizational support	40	120	88.99	23.23	0.23 **	−0.09	0.04	−0.05	−0.03	0.39 *				
8. OCB change as Transfer 1	−14	38	6.90	9.29	0.67 **	0.04	−0.08	0.03	−0.05	0.53 **	0.22 **			
9. OCB change as Transfer 2	14	33	6.14	8.75	0.62 **	0.02	0.09	0.03	−0.03	0.49 **	0.27 **	0.95 **		
10. CWB change as Transfer 1	−41	19	−5.00	8.96	−0.48	−0.04	0.14	−0.11	0.04	−0.52 **	−0.19 **	−0.48 **	−0.46 **	
11. CWB change as Transfer 2	−38	12	−5.00	8.62	−0.41	−0.03	0.10	−0.10	0.01	−0.45 **	−0.11	−0.43 **	−0.38 **	0.87 **
**(b) Supervisor Evaluation Sample (** ***n* =** **78)**														
	**Minimum**	**Maximum**	**Mean**	**SD**	**1**	**2**	**3**	**4**	**5**	**6**	**7**	**8**		
1. Training	0	1	0.63	0.48										
2. Age	27	54	40.13	5.88	0.11									
3. Gender	1	2	1.13	0.33	−0.02	−0.21								
4. No. years in public service	1	29	12.22	6.68	0.08	0.70 **	−0.15							
5. Educational qualifications	1	3	2.29	0.60	−0.02	0.21	−0.06	0.38 **						
6. Learning	−14	48	10.51	12.95	0.55 **	0.22 *	−0.04	0.25 *	0.01					
7. Social and organizational support	42	120	89.69	22.22	0.28 *	−0.07	−0.01	0.05	−0.01	0.41 **				
8. OCB change as Transfer 2	−34	56	9.77	15.07	0.53 **	0.03	0.10	0.10	0.10	0.184	−0.01			
9. CWB change as Transfer 2	−60	62	−9.88	23.82	−0.28 *	−0.09	−0.01	−0.21	−0.13	−0.07	0.08	−0.33 **		

Note: Age was measured in a 10-year window starting at 20 years. Sex was coded as 1 = female, 2 = male, and workplace was coded as 1 = working in the headquarters of the WPC, and 2 = working in other affiliated institutions. No. years of public service were measured in 10-year windows starting from 1 year. Educational qualification was coded as 3 = master’s degree, 2 = bachelor’s degree, and 1 = high school. Transfer 1 = behavior changes between before and 1 month after the training. Transfer 2 = behavior changes between before and 3 months after the training, SD = standard deviation, *n* = sample size. * *p* < 0.05, ** *p* < 0.01.

**Table 4:** Results of the reliability test and convergent validity tests.

Construct	Self-Evaluation Sample (*n* = 176)		Supervisor Evaluation Sample (*n* = 78)	
	Cronbach’s Alpha	AVE Value	Cronbach’s Alpha	AVE Value
Ability level before the training	0.918	0.502	0.826	0.628
Ability level just after the training	0.941	0.501	0.924	0.560
Social and organizational support	0.950	0.627	0.934	0.501
OCB level before the training	0.847	0.503	0.908	0.538
CWB level before the training	0.944	0.599	0.981	0.501
OCB level at 1 month after training	0.920	0.654		
CWB level at 1 month after training	0.957	0.539		
OCB level at 3 months after training	0.897	0.672	0.919	0.630
CWB level at 3 months after training	0.954	0.501	0.979	0.677

Note: *n* = sample size.

**Table 5:** Results of discriminant validity tests of the measurements in the (a) self-evaluation sample and (b) supervisor evaluation sample.

(a) Self-Evaluation Sample (*n* = 176)									
Component	1	2	3	4	5	6	7	8	9
1.Ability level before the training	0.708								
2. Ability level just after the training	−0.314	0.707							
3. Social and organization support	0.142	0.018	0.792						
4. OCB level before the training	0.259	−0.126	−0.219	0.709					
5. CWB level before the training	0.691	−0.194	−0.029	0.393	0.774				
6. OCB level at 1 month after training	−0.603	0.226	−0.149	−0.578	−0.376	0.809			
7. CWB level at 1 month after training	−0.130	0.833	0.105	−0.268	−0.223	0.098	0.734		
8. OCB level at 3 months after training	−0.068	−0.037	−0.090	0.013	−0.013	0.046	−0.042	0.820	
9. OCB level at 3 months after training	0.005	0.002	−0.006	0.053	0.049	0.035	−0.050	−0.119	0.707
**(b) Supervisor Evaluation Sample (*n* = 78)**									
**Component**	**1**	**2**	**3**	**4**	**5**	**6**	**7**		
1. Learning before the training	0.792								
2. Learning just after the training	0.099	0.748							
3. Social and organization support	0.144	−0.151	0.707						
4. OCB level before the training	0.118	0.015	0.106	0.734					
5. CWB level before the training	−0.470	0.196	−0.177	−0.265	0.707				
6. OCB level at 3 months after training	0.418	0.464	0.276	0.179	−0.019	0.794			
7. CWB level at 3 months after training	0.633	0.144	0.093	0.095	−0.214	0.516	0.823		

Note: *n* = sample size.

**Table 6:** Result of Independent Samples *t*-test of ability test scores on EI between Treatment and Control Groups at Time (0) and Time (1).

Time Frame	Group		Mean	SD	MD	St. Error_ Difference	95% Confidence Interval of the Difference		*p*
							Lower	Upper	
Ability level at Time (0)	Treatment	132.08		15.904	1.545	2.210	−2.815	5.907	0.485
	Control	130.53		13.294					
Ability level at Time (1)	Treatment	148.17		12.241	17.557	1.823	13.957	21.55	0.000
	Control	130.61		11.949					
Learning	Treatment	16.09		13.373	16.011	1.655	12.744	19.278	0.000
	Control	0.08		7.889					

Note: Time (0) = before training, Time (1) = immediately after training, SD = standard deviation, MD = mean difference.

**Table 7:** Result of Independent Samples *t*-test of OCB and CWB Change in Self- and Supervisor Evaluation Samples’ Analysis at Time (0), Time (2), Time (3), Transfer 1, and Transfer 2.

Time Frame	Group	Mean	SD	MD	St. Error_ Difference	95% Confidence Interval of the Difference		*p*
						Lower	Upper	
Self-Evaluation Sample on OCB								
OCB level at Time (0)	Treatment	145.90	10.283	1.120	1.663	−2.282	4.282	0.548
	Control	144.88	11.733					
OCB level at Time (2)	Treatment	159.08	8.453	13.557	1.521	10.555	16.557	0.000
	Control	145.52	11.489					
OCB level at Time (3)	Treatment	157.51	8.658	11.955	1.506	8.981	14.927	0.000
	Control	145.56	11.166					
OCB change as Transfer 1 (Time (2)-(0))	Treatment	13.180	7.630	12.557	1.033	10.517	14.596	0.000
	Control	0.630	5.978					
OCB change as Transfer 2 (Time (3)-(0))	Treatment	11.61	7.747	10.955	1.035	8.912	12.996	0.000
	Control	0.66	5.845					
Self-Evaluation Sample on CWB								
CWB level at Time (0)	Treatment	61.35	11.472	−0.750	2.348	−5.383	3.883	0.750
	Control	62.10	18.801					
CWB level at Time (2)	Treatment	52.03	6.669	−9.375	2.046	−13.413	−5.336	0.000
	Control	61.41	17.998					
CWB level at Time (3)	Treatment	52.77	6.683	−7.898	1.992	−11.828	−3.966	0.000
	Control	60.67	17.447					
CWB change as Transfer 1 (Time (2)-(0))	Treatment	−9.31	8.640	−8.614	1.188	−10.958	−6.269	0.000
	Control	−0.69	7.037					
CWB change as Transfer 2 (Time (3)-(0))	Treatment	−8.57	8.854	−7.136	1.186	−9.477	−4.795	0.000
	Control	−1.43	6.738					
Supervisor Evaluation Sample on OCB								
OCB level at Time (0)	Treatment	136.31	12.361	0.651	2.635	−4.598	5.899	0.806
	Control	135.66	9.025					
OCB level at Time (3)	Treatment	152.18	6.116	17.080	1.786	13.524	20.636	0.000
	Control	135.10	9.671					
OCB change as Transfer 2 (Time (3)-(0))	Treatment	15.88	13.319	16.429	3.015	10.424	22.434	0.000
	Control	−0.55	12.058					
Supervisor Evaluation Sample on CWB								
CWB level as Time (0)	Treatment	87.04	27.045	−5.890	6.121	−18.081	6.300	0.339
	Control	92.93	24.469					
CWB level at Time (3)	Treatment	71.94	11.621	−19.923	3.655	−27.202	−12.645	0.000
	Control	91.86	20.710					
CWB change as Transfer 2 (Time (3)-(0))	Treatment	−15.10	25.887	−14.033	5.383	−24.755	−3.312	0.011
	Control	−1.07	16.856					

Note: Time (0) = before the training, Time (2) = 1 month after the training, Time (3) = 3 months after the training, OCB = organizational citizenship behavior, CWB = counterproductive work behavior, SD = standard deviation, Transfer 1 = behavior before the training and behavior change 1 month after the training, Transfer 2 = behavior before and behavior change 3 months after the training, MD = mean difference.

**Table 8:** Multiple Group Comparison Test for OCB and CWB.

Multiple Group Comparison Test for OCB						
(I) Group		MD	Std. Error	Sig	95% Confidence Interval	
					Lower Bound	Upper Bound
1	2	−13.182 *	1.419	0.000	−17.39	−8.97
	3	−11.614 *	1.433	0.000	−15.87	−7.36
	4	1.000	1.663	1.000	−3.93	5.93
	5	0.375	1.644	1.000	−4.50	5.25
	6	0.341	1.618	1.000	−4.46	5.14
2	3	1.568	1.290	0.976	−2.26	5.39
	4	14.182 *	1.542	0.000	9.60	18.76
	5	13.557 *	1.521	0.000	9.04	18.07
	6	13.523 *	1.493	0.000	9.09	17.96
3	4	12.614 *	1.554	0.000	8.00	17.23
	5	11.989 *	1.534	0.000	7.43	16.54
	6	11.955 *	1.506	0.000	7.48	16.43
4	5	−0.625	1.751	1.000	−5.82	4.57
	6	−0.659	1.727	1.000	−5.78	4.46
5	6	−0.034	1.708	1.000	−5.10	5.03
Multiple Group Comparison Test for CWB						
(I) Group		MD	Std. Error	Sig.	95% Confidence Interval	
					Lower Bound	Upper Bound
1	2	9.318 *	1.415	0.000	5.11	13.53
	3	8.580 *	1.415	0.000	4.37	12.79
	4	−0.750	2.348	1.000	−7.73	6.23
	5	−0.057	2.275	1.000	−6.82	6.71
	6	0.682	2.226	1.000	−5.93	7.30
2	3	−0.739	1.006	1.000	−3.72	2.25
	4	−10.068 *	2.127	0.000	−16.43	−3.71
	5	−9.375 *	2.046	0.000	−15.49	−3.26
	6	−8.636 *	1.991	0.000	−14.59	−2.69
3	4	−9.330 *	2.127	0.000	−15.69	−2.97
	5	−8.636 *	2.047	0.001	−14.75	−2.52
	6	−7.898 *	1.992	0.002	−13.85	−1.95
4	5	0.693	2.775	1.000	−7.54	8.92
	6	1.432	2.734	1.000	−6.68	9.54
5	6	0.739	2.672	1.000	−7.19	8.67

Note: Group 1 = before the training treatment group; Group 2 = 1 month after the training treatment group; Group 3 = 3 months after the training treatment group; Group 4 = before the training control group; Group 5 = 1 month after the training control group; Group 6 = 3 months after the training control group. MD = mean difference. The MD was significant at the level of 0.05. * *p* < 0.05.

**Table 9:** Regression Coefficient, Standard Error, and Model Summary Information for Mediation Model of OCB and CWB change as Transfer 1 and Transfer 2 of Self- and Supervisor Evaluation Samples.

Self-Evaluation Sample: OCB Change as Transfer 1						
	Learning			Transfer		
	Coeff.	SE	*p*	Coeff.	SE	*p*
Training	15.951	1.678	0.000	10.384	1.269	0.000
Learning				0.139	0.047	0.000
Constant	2.753	8.141	0.736	−2.453	4.969	0.622
	R^2^ = 0.361			R^2^ = 0.469		
	F (6, 169) = 15.941, *p* = 0.000			F (7, 168) = 23.573, *p* = 0.000		
Self-Evaluation Sample: OCB Change as Transfer 2						
	Learning			Transfer		
	Coeff.	SE	*p*	Coeff.	SE	*p*
Training	15.951	1.678	0.000	9.111	1.277	0.000
Learning				0.120	0.047	0.011
Constant	2.753	8.141	0.736	−1.560	5.003	0.755
	R^2^ = 0.361			R^2^ = 0.426		
	F (6, 169) = 15.941, *p* = 0.000			F (7, 168) =17.853, *p* = 0.000		
Supervisor Evaluation Sample: OCB Change as Transfer 2						
	Learning			Transfer		
	Coeff.	SE	*p*	Coeff.	SE	*p*
Training	14.569	2.618	0.000	10.296	3.729	0.000
Learning				−0.212	0.141	0.138
Constant	−6.407	14.622	0.000	0.879	17.363	0.959
	R^2^ = 0.363			R^2^ = 0.348		
	F (8, 69) = 4.618, *p* = 0.000			F (7, 70) = 5.709, *p* = 0.000		
Self-Evaluation Sample: CWB Change as Transfer 1						
	Learning			Transfer		
	Coeff.	SE	*p*	Coeff.	SE	*p*
Training	15.951	1.678	0.000	−4.773	1.393	0.000
Learning				−0.234	0.051	0.000
Constant	2.752	8.141	0.735	−7.304	5.457	0.182
	R^2^ = 0.361			R^2^ = 0.346		
	F (6, 169) =15.940, *p* = 0.000			F (7, 168) =12.725, *p* = 0.000		
Self-Evaluation Sample: CWB Change as Transfer 2						
	Learning			Transfer		
	Coeff.	SE	*p*	Coeff.	SE	*p*
Training	15.951	1.678	0.000	−3.898	1.421	0.006
Learning				−0.200	0.052	0.000
Constant	2.752	8.141	0.735	−5.202	5.569	0.360
	R^2^ = 0.361			R^2^ = 0.264		
	F (6, 169) = 15.940, *p* = 0.000			F (7, 168) = 8.609, *p* = 0.000		
Supervisor Evaluation Sample: CWB Change as Transfer 2						
	Learning			Transfer		
	Coeff.	SE	*p*	Coeff.	SE	*p*
Training	14.569	2.618	0.000	−20.413	6.517	0.002
Learning				0.407	0.247	0.109
Constant	−6.407	14.622	0.662			
	R^2^ = 0.363			R^2^ = 0.203		
	F (7, 70) = 5.709, *p* = 0.000			F (8, 69) = 2.200, *p* = 0.037		

Note: Coefficient, unstandardized regression coefficient; SE, standard error; *p*, *p*-value; *n*, number of participants.

**Table 10:** Results of Mediation Analysis.

Self-Evaluation Sample					
Relationship	Total Effect	Direct Effect	Indirect Effect	95% Confidence Interval	
				Lower Bound	Upper Bound
Training > Learning > OCB change as Transfer 1	12.599 ***	10.384 ***	2.215	0.948	4.015
Training > Learning > OCB change as Transfer 2	11.029 ***	9.111 ***	1.917	0.699	3.596
Training > Learning > CWB change as Transfer 1	−8.507 ***	−4.773 ***	−3.734	−5.686	−1.579
Training > Learning > CWB change as Transfer 2	−7.094 ***	−3.899 **	−3.195	−4.997	−1.164
Supervisor Evaluation Sample					
Training > Learning > OCB change as Transfer 2	16.199 ***	19.296 ***	−3.097	−7.308	1.537
Training > Learning > CWB change as Transfer 2	−14.560 ***	−20.413 ***	5.853	−0.952	13.887

Note: ** *p* < 0.01, *** *p* < 0.001.

**Table 11:** Results of Hierarchical Regression Analysis: Exploring the Moderating Effect of Social and Organizational Support on the Relationship between Training and Learning.

Self-Evaluation Sample				
	Model A (without Moderation)		Model B (with Moderation)	
	Standardized Coefficients	*t*-Value	Standardized Coefficients	*t*-Value
Step 1				
Training	0.508 ***	8.459	0.472 ***	8.971
Social and organizational support	0.289 ***	4.819	0.452 ***	7.971
Step 2				
Training × Social and organizational support			0.413 ***	7.448
R^2^ change	0.405		0.145	
F change	58.954 ***		55.473 ***	

Note: *** *p* < 0.001.

**Table 12:** Results of Hierarchical Regression Analysis: Exploring the Moderating Effect of Social and Organizational Support on the Relationship between Learning and OCB and CWB Change as Transfer 1 and Transfer 2 of Self- and Supervisor Evaluation.

Self-Evaluation Sample: Moderation Analysis on the Relationship between Learning and OCB Change as Transfer 1				
	Model A (Without Moderation)		Model B (With Moderation)	
	Standardized Coefficients	*t*-Value	Standardized Coefficients	*t*-Value
Step 1				
Learning	0.525 ***	7.467	0.610 ***	5.449
Social and organizational support	0.014	0.202	−0.027	−0.325
Step 2				
Learning * Social and organizational support			−0.101	−0.977
R^2^ change	0.282		0.004	
F change	33.90 ***		0.955	
Self-Evaluation Sample: Moderation Analysis on the Relationship between Learning and OCB Change as Transfer 2				
	Model A (Without Moderation)		Model B (With Moderation)	
	Standardized Coefficients	*t*-Value	Standardized Coefficients	*t*-Value
Step 1				
Learning	0.481 ***	6.663	0.548 ***	4.758
Social and organizational support	0.025	0.343	−0.007	−0.085
Step 2				
Learning * Social and organizational support			−0.079	−0.743
R^2^ change	0.242		0.002	
F change	27.562 ***		0.552	
Self-Evaluation Sample: Moderation Analysis on the Relationship between Learning and CWB Change as Transfer 1				
	Model A (Without Moderation)		Model B (With Moderation)	
	Standardized Coefficients	*t*-Value	Standardized Coefficients	*t*-Value
Step 1				
Learning	−0.529 ***	−7.481	−0.397 ***	−3.544
Social and organizational support	0.016	0.224	−0.047	−0.576
Step 2				
Learning * Social and organizational support			−0.155	−1.505
R^2^ change	0.273		0.009	
F change	32.518 ***		2.266	
Self-Evaluation Sample: Moderation Analysis on the Relationship between Learning and CWB Change as Transfer 2				
	Model A (Without Moderation)		Model B (With Moderation)	
	Standardized Coefficients	*t*-Value	Standardized Coefficients	*t*-Value
Step 1				
Learning	−0.494 ***	−6.726	−0.389 ***	−3.328
Social and organizational support	0.089	1.216	−0.039	0.455
Step 2				
Learning * Social and organizational support			−0.124	−1.157
R^2^ change	0.217		0.006	
F change	23.904 ***		1.340	
Supervisor Evaluation Sample: Moderation Analysis on the Relationship between Learning and OCB Change as Transfer 2				
	Model A (Without Moderation)		Model B (With Moderation)	
	Standardized Coefficients	*t*-Value	Standardized Coefficients	*t*-Value
Step 1				
Learning	0.228	1.833	0.339 *	2.169
Social and organizational support	−0.105	−0.842	−0.171	−1.251
Step 2				
Learning * Social and organizational support			−0.167	−1.167
R^2^ change	0.043		0.017	
F change	1.84		1.362	
Supervisor Behavior Evaluation: Moderation Analysis on the Relationship between Learning and CWB Change as Transfer 2				
	Model A (Without Moderation)		Model B (With Moderation)	
	Standardized Coefficients	*t*-Value	Standardized Coefficients	*t*-Value
Step 1				
Learning	−0.127	−1.009	−0.171	−1.076
Social and organizational support	−0.137	1.087	0.163	1.174
Step 2				
Learning * Social and organizational support			0.067	0.459
R^2^ change	0.020		0.003	
F change	0.777		0.210	

Note: * *p* < 0.05, *** *p* < 0.001.

**Table 13:** Results of Hierarchical Regression Analysis: Exploring the Moderating Effect of Social and Organizational Support on the Relationship between Training and OCB and CWB Change as Transfer 1 and Transfer 2 of the Self-Evaluation Sample.

Self-Evaluation Sample: Moderation Analysis on the Relationship between Training and OCB Change as Transfer 1				
	Model A (Without Moderation)		Model B (With Moderation)	
	Standardized Coefficients	*t*-Value	Standardized Coefficients	*t*-Value
Step 1				
Training	0.641 ***	11.008	0.622 ***	11.002
Social and organizational support	0.085	1.465	0.170 **	2.790
Step 2				
Training * Social and organizational support			0.215 ***	3.611
R^2^ change	0.441		0.039	
F change	68.334 ***		13.042 ***	
Self-Evaluation Sample: Moderation Analysis on the Relationship between Training and OCB Change as Transfer 2				
	Model A (Without Moderation)		Model B (With Moderation)	
	Standardized Coefficients	*t*-Value	Standardized Coefficients	*t*-Value
Step 1				
Training	0.585 ***	9.479	0.567 ***	9.390
Social and organizational support	0.091	1.468	0.170 **	2.617
Step 2				
Training * Social and organizational support			0.202 **	3.184
R^2^ change	0.373		0.035	
F change	51.407 ***		10.136 **	
Self-Evaluation Sample: Moderation Analysis on the Relationship between Training and CWB Change as Transfer 1				
	Model A (Without Moderation)		Model B (With Moderation)	
	Standardized Coefficients	*t*-Value	Standardized Coefficients	*t*-Value
Step 1				
Training	−0.433 ***	−6.285	−0.412 ***	−6.131
Social and organizational support	−0.102	−1.475	−0.195 **	−2.691
Step 2				
Training * Social and organizational support			−0.237 **	−3.347
R^2^ change	0.217		0.048	
F change	23.963 ***		11.200 *	
Self-Evaluation Sample: Moderation Analysis on the Relationship between Training and CWB Change as Transfer 2				
	Model A (Without Moderation)		Model B (With Moderation)	
	Standardized Coefficients	*t*-Value	Standardized Coefficients	*t*-Value
Step 1				
Training	−0.390 ***	−5.456	−0.367 ***	−5.285
Social and organizational support	−0.023	−0.328	−0.127	−1.690
Step 2				
Training * Social and organizational support			−0.261 ***	−3.571
R^2^ change	0.157		0.058	
F change	16.076 ***		12.750 ***	

Note: * *p* < 0.05, ** *p* < 0.01, *** *p* < 0.001.

## Data Availability

The data presented in this study will be openly available at Mendeley Data; https://doi.org/10.17632/f4tbc642bj.1.

## Supplementary Materials

The following supporting information can be downloaded at: https://www.mdpi.com/article/10.3390/bs14040276/s1, Table S1: Means, Standard Deviation, and Intercorrelations of Variables in both Samples with all the variables, (a) Self-evaluation sample (*n* = 176), (b) Supervisor-evaluation sample (*n* = 78); Table S2: Non-response Bias Analysis for Supervisors Sample Before the Training and After 3 Months of the Training; Table S3: Balance Check Between the Treatment and Control Groups Before the training; Table S4: Results of Hierarchical Regression Analysis, Examining the Moderating Effect of Social and Organizational Support on the Relationship between Training and OCB and CWB at Transfer 2 of the Supervisor Evaluation.

## Appendix A

Measurement Items

Ability level (Schutte et al. [150])

1. I know when to speak about my problems to others.
2. When I am faced with obstacles, I remember times I faced similar obstacles and overcame them.
3. I expect that I will do well in most things I try.
4. Other people find it easy to confide in me.
5. I find it hard to understand the non-verbal messages of other people.
6. Some of the major events of my life have led me to re-evaluate what is important and not important.
7. When my mood changes, I see new possibilities.
8. Emotions are one of the things that make my life worth living.
9. I am aware of my emotions as I experience them.
10. I expect good things to happen.
11. I like to share my emotions with others.
12. When I experience a positive emotion, I know how to make it last.
13. I arrange events others enjoy.
14. I seek out activities that make me happy.
15. I am aware of the non-verbal messages I send to others.
16. I present myself in a way that makes a good impression on others.
17. When I am in a positive mood, solving problems is easy for me.
18. By looking at their facial expressions, I recognize the emotions people are experiencing.
19. I know why my emotions change.
20. When I am in a positive mood, I can come up with new ideas.
21. I have control over my emotions.
22. I easily recognize my emotions as I experience them.
23. I motivate myself by imagining a good outcome for the tasks I take on.
24. I compliment others when they have done something well.
25. I am aware of the non-verbal messages other people send.
26. When another person tells me about an important event in his or her life, I almost feel as though I have experienced this event myself.
27. When I feel a change in emotions, I tend to come up with new ideas.
28. When I am faced with a challenge, I give up because I believe I will fail.
29. I know what other people are feeling just by looking at them.
30. I help other people feel better when they are down.
31. I use good moods to help myself keep trying in the face of obstacles.
32. I can tell how people are feeling by listening to the tone of their voice.
33. It is difficult for me to understand why people feel the way they do.

Organizational Citizenship Behavior (Podsakoff et al. [159])

1. Help others who have a heavy workload.
2. Always ready to lend a helping hand to those around me.
3. Help others who have been absent.
4. Willing to help others who have work-related problems.
5. Help orient new people even though it is not required.
6. One of the most conscientious employees.
7. Believe in giving an honest day’s work for an honest day’s pay.
8. Attendance at work is above the norm.
9. Do not take extra breaks.
10. Obey company rules and regulations even when no one is watching.
11. Not the classic “squeaky wheel” that always needs greasing.
12. Do not spend a lot of time complaining about trivial matters.
13. Do not tend to make “Mountains out of molehills”.
14. Always focus on the positive side rather than the negative side.
15. Never find fault with what the organization is doing.
16. Try to avoid creating problems for co-workers.
17. Consider the impact of actions on coworkers.
18. Do not abuse the rights of others.
19. Take steps to try to prevent problems with other workers.
20. Mindful of how behavior affects other people’s jobs.
21. Keep abreast of changes in the organization.
22. Attend meetings that are not mandatory but are considered important.
23. Attend functions that do not require the help of the company image.
24. Read and keep up with organization announcements, memos and so on.

Counterproductive Work Behavior (Spector et al. [130])

1. Purposely wasted employer’s materials/supplies/resources.
2. Daydreamed rather than did the work.
3. Complained about insignificant things at work.
4. Told people outside the job what a lousy place they work for.
5. Purposely did work incorrectly.
6. Came to work late without permission.
7. Stayed home from work and said sick when not.
8. Purposely damaged a piece of equipment or property.
9. Purposely dirtied or littered the place of work.
10. Stolen something belonging to the employer.
11. Started or continued a damaging or harmful rumour at work.
12. Being nasty or rude to a client or customer.
13. Purposely worked slowly when things needed to get done.
14. Refused to take on an assignment when asked.
15. Purposely came late to an appointment or meeting.
16. Failed to report a problem so it would get worse.
17. Taken a longer break than allowed to take.
18. Purposely failed to follow instructions.
19. Left work earlier than allowed to.
20. Insulted someone about their job performance.
21. Made fun of someone’s personal life.
22. Took supplies or tools home without permission.
23. Tried to look busy while doing nothing.
24. Put in to be paid for more hours than worked.
25. Took money from the employer without permission.
26. Ignored someone at work.
27. Refused to help someone at work.
28. Withheld needed information from someone at work.
29. Purposely interfered with someone at work doing his/her job.
30. Blamed someone at work for what made.
31. Started an argument with someone at work.
32. Stolen something belonging to someone at work.
33. Verbally abused someone at work.
34. Made an obscene gesture (the finger) to someone at work.
35. Threatened someone at work with violence.
36. Threatened someone at work‚ but not physically.
37. Said something obscene to someone at work to make them feel bad.
38. Hidden something so someone at work can’t find it.
39. Did something to make someone at work look bad.
40. Played a mean prank to embarrass someone at work.
41. Destroyed property belonging to someone at work.
42. Looked at someone at work’s private mail/property without permission.
43. Hit or pushed someone at work.
44. Insulted or made fun of someone at work.
45. Avoided returning a phone call to someone who should be at work.

Social and Organizational Support (Holton et al. [160] and Kupritz [161])

Social Support

1. My supervisor helps me set goals for applying new KSA.
2. My supervisor sets the criteria for applying new KSA to my job.
3. My supervisor assists me when I have a problem trying out KSA.
4. My supervisor discusses how to apply KSA to job situations.
5. My supervisor informs me how well I accomplish tasks by using KSA.
6. My supervisor informs me of our group’s performance in accomplishing tasks.
7. My peers help me with information in applying for a new KSA.
8. My peers care about my applying for the new KSA on the job.

Organizational Support

1. Workplace design features supporting privacy needs.
2. My organization has a strategy plan and is interested in the personal and professional development of employees.
3. Proximity close to direct reports.
4. Sharing of workspace to increase communication.
5. Aesthetically pleasing, with adequate comfort level, the corporate image reflected in the workspace.
6. Workspace with Windows.
7. Effective interpersonal communication.
8. Efficient management of logistic resources.
9. Efficient management of human resources.
10. Workplace design features do not support privacy needs.
11. My organization has inefficient and inflexible workspace for teaching knowledge and skills from training to other employees.
12. Proximity is too far away from direct reports.
13. Space is not shared, creating communication barriers.
14. Ineffective interpersonal communication.
15. Inefficient management of logistic resources.
16. Inefficient management of human resources.
