# Supplementary material for: Does Social and Organizational Support Moderate Emotional Intelligence Training Effectiveness?
Source: Behav Sci (Basel). 2024 Mar 26;14(4):276. doi: 10.3390/bs14040276 (PMC11047385; doi:10.3390/bs14040276)
Supplement: Supplementary file 1 [file behavsci-14-00276-s001.zip › behavsci-2857860-supplementary.pdf]

behavioral sciences

MDPI

Article

Does Social and Organizational Support Moderate Emotional Intelligence Training Effectiveness?

Ishara Madhunika Opatha and Yoshi Takahashi *

Graduate School of Humanities and Social Sciences, Hiroshima University, Higashihiiroshima 739-8529, Japan; d212441@hiroshima-u.ac.jp

* Correspondence: yoshit@hiroshima-u.ac.jp

Tables

Table S1. Means, Standard Deviation, and Intercorrelations of Variables in both Samples with all the variables. (a)

	Minimum	Maximum	Mean	SD	1	2	3	4	5	6	7	8	9	10	11	12	13	14	15	16	17	18
**1**	0	1	0.5	0.51																		
**2**	27	55	39	6	.06																	
**3**	1	2	1	.32	-.02	-.03																
**4**	1	30	12	7	.05	.716**	-.07															
**5**	1	3	2	1	-.09	.253**	.07	.388**														
**6**	58	163	131	15	.05	-.06	-.16*	.03	.01													
**7**	74	165	139	15	.590**	.00	-.16*	.13	-.01	.57**												
**8**	-19	80	8	14	.591**	.06	.01	.11	-.02	-.44**	.47**											
**9**	40	120	89	23	.231**	-.09	.04	-.05	-.03	-.21**	0.1	.39*										
**10**	102	166	145	11	.04	-.08	-.28	-.05	.02	.39**	.37**	-.01	.03									
**11**	102	168	152	12	.56**	-.04	-.19	-.03	-.02	.25**	.60**	.397**	.14	.68**								
**12**	104	168	152	12	.51**	-.05	-.19	-.03	.00	.27**	.60**	.361**	.14	.70**	.97**							
**13**	-14	38	7	9	.67**	.04	-.08	.03	-.05	-0.1	.35**	.530**	.22**	-.29**	.49**	.44**						
**14**	-14	33	6	9	.62**	.02	.09	.03	-.03	-0.1	.32**	.491**	.27**	-.32**	.43**	.44**	.95**					
**15**	45	159	62	16	-.02	.06	.12	.10	.02	-0.2	-.10	.180*	.10	-.22**	-.12	-.14*	0.10	.08				
**16**	45	156	57	14	-.32	.04	.21**	.04	.04	-0.1	-.25**	-.13	-.01	-.21**	-.34**	-.35**	-.19*	-.20**	.82**			
**17**	45	149	57	14	-.28	.05	.19**	.05	.02	-0.1	-.21**	-.08	.05	-.19*	-.29**	-.29**	-.15*	-.14*	.83**	.95**		
**18**	-41	19	-5	9	-.48	-.04	.14	-.11	.04	.24**	-.23**	-.523**	-.19**	.04	-.33**	-.31**	-.48**	-.46**	-.41**	.17*	.08	
**19**	-38	12	-5	9	-.41	-.03	.10	-.10	.01	.26**	-.15*	-.458**	-.11	.09	-.24**	-.20**	-.43**	-.38**	-.47**	.04	.09	.87**

Note: Age was measured in a 10-year window starting at 20 years. Sex was coded as 1= female, 2= male, and workplace was coded as 1=working in the headquarters of the WPC, and 2=working in other affiliated institutions. No years of public service were measured in the 10-year window starting from 1 year. Educational qualification was coded as 3= master's degree levels, 2-degree levels, and 1= high school. Transfer1 behavior changes before and 1 month after the training. Transfer

2= behavioral changes before and 3 months after the training, SD = Standard deviation, N=sample size. 1=Training, 2=Age, 3=Gender, 4=.No of years in the public service, 5= Educational Qualifications 6= ability level before training (ability test score),7= ability level just after the training (ability test score), 8=learning (ability test score before and after),9=social and organizational support, 10= OCB level before the training ,11= OCB level after 1 month, 12=OCB level after 3 months,13=OCB change before and 1month (Transfer 1), 14=OCB change before and after 3 months, 15=CWB level before the training, 16= CWB level after 1 month, 17=CWB level after 3 months,18=CWB change before and 1month (Transfer 1), 19=CWB change before and after 3 months, *p < 0.05, **p < 0.01, ***p < 0.001.

Table S1. Means, Standard Deviation, and Intercorrelations of Variables in both Samples with all the variables. (b)

	Minimum	Maximum	Mean	SD	1	2	3	4	5	6	7	8	9	10	11	12	13	14
**1**	0	1	0.63	.48														
**2**	27	54	40.13	5.88	.11													
**3**	1	2	1.13	.33	-.02	-.21												
**4**	1	29	12.22	6.68	.08	.70**	-.15											
**5**	1	3	2.29	.60	-.02	.21	-.06	.38**										
**6**	106	163	132.05	10.96	.07	-.09	-.02	-.04	-.13									
**7**	114	165	142.56	13.65	.58**	.13	-.05	.19	-.09	.46*								
**8**	-14	48	10.51	12.95	.55**	.22*	-.042	.25*	.01	-.35**	.66**							
**9**	42	120	89.69	22.22	.28*	-.07	-.01	.05	-.01	-.15	.27*	.41**						
**10**	100	167	136.06	11.17	.02	.14	-.01	-.01	-.14	.01	.17	.17	.10					
**11**	115	164	145.83	11.24	.73**	.18	.13	.12	-.01	.14	.51**	.42**	.09	.09				
**12**	-34	56	9.77	15.07	.53**	.03	.10	.10	.10	.09	.25*	.184	-.01	-.67**	.67**			
**13**	45	129	89.23	26.11	-.11	.06	-.01	.15	.13	.19	-.01	-.186	-.17	-.26*	-.21	.03		
**14**	46	116	79.35	18.27	-.53**	-.03	-.02	-.05	.02	-.09	-.41**	-.35**	-.14	-.11	-.63*	-.38**	.47**	
**15**	-60	62	-9.88	23.82	-.28*	-.09	-.01	-.21	-.13	-.29**	-.30**	-.07	.08	.19	-.25*	-.33**	-.73**	.25*

Note: Age was measured in a 10-year window starting at 20 years. Sex was coded as 1= female, 2= male, and workplace was coded as 1=working in the headquarters of the WPC, and 2=working in other affiliated institutions. No years of public service were measured in the 10-year window starting from 1 year. Educational

qualification was coded as 3= master's degree levels, 2-degree levels, and 1= high school. Transfer1 behavior changes before and 1 month after the training. Transfer 2= behavioral changes before and 3 months after the training, SD = Standard deviation, N=sample size. 1=Training, 2=Age, 3=Gender, 4=.No of years in the public service, 5= Educational Qualifications 6= Ability level before training (ability test score),7= ability level just after training(ability test score), 8=learning (ability test score before and after),9=social and organizational support, 10= OCB level before the training,11=OCB level at after 3 months,12=OCB change before and after 3 months (Transfer 2), 13=CWB level before the training, 14= CWB level at after 3 months,15= CWB change before and after 3 months, *p < 0.05, **p < 0.01, ***p < 0.001.

Table S2. Non-response Bias Analysis for Supervisors Sample Before the Training and After Three Months of Training. **Chi-square test results for Categorical variables**

		**Non-response bias check after data collection before the Training**			**Non-response bias check after data collection after three months of the training**		
**Variable**	**Group**	**Mean**	**Chi-Square value**	***p***	**Mean**	**Chi-Square value**	***p***
Gender	Responded Supervisors	1.93	.377	.539	1.88	1.451	0.228
	Non-Responded Supervisors	2.00			2.00		
Educational Qualification	Responded Supervisors	1.50	1.360	.243	1.38	2.358	.125
	Non-Responded Supervisors	1.80			1.73		
Position	Responded Supervisors	2.14	.827	.661	2.00	.344	.842
	Non-Responded Supervisors	1.80			2.09		
Workplace	Responded Supervisors	1.43	2.039	.153	1.63	.540	.463
	Non-Responded Supervisors	1.80			1.45		

Independent Sample T-test results for continuous variables

	**Non-response bias check after data collection before the Training**			**Non-response bias check after data collection after three months of the training**	
**Group**	**Mean**	**MD**	***p***	**Mean**	**MD** ***p***

Age	Responded Supervisors	44.00	1.000	.683	45.00	2.182	.311
	Non-Responded Supervisors	43.00			42.82		
No years in the Public Service	Responded Supervisors	16.21	3.414	.232	16.63	2.261	.379
	Non-Responded Supervisors	12.80			14.36		

MD=mean difference.

Table S3. Balance Check Between the Treatment and Control Groups Before the training.

Chi-square test results for Categorical variables

Variable	Group	Mean	Chi-Square value	p
**Self-Evaluation Sample**				
Workplace	Treatment	2.27	.024	.878
	Control	2.38		
Gender	Treatment	1.11	.054	.816
	Control	1.13		
Educational Qualification	Treatment	2.27	1.384	.501
	Control	2.38		
**Supervisor Evaluation Sample**				
Workplace	Treatment	1.36	.203	.652
	Control	1.24		
Gender	Treatment	1.16	2.121	.143
	Control	1.13		
Educational Qualification	Treatment	2.31	.424	.809
	Control	2.35		

Independent Sample T-test results for continuous variables

Variable	Group	Mean	MD	p

**Self-Evaluation Sample**				
Age	Treatment	39.84	.693	.446
	Control	39.15		
No years in the Public Service	Treatment	12.03	.648	.544
	Control	11.39		
**Supervisor_-Evaluation Sample**				
Age	Treatment	40.67	1.090	.340
	Control	39.58		
No years in the Public Service	Treatment	12.33	-.035	.978
	Control	12.36		

Note: MD=mean difference

Table S4. Results of Hierarchical Regression Analysis, Examining the Moderating Effect of Social and Organizational Support on the Relationship between Training and OCB and CWB at Transfer 2 of the Supervisor Evaluation.

Supervisor Evaluation Sample: Moderation Analysis on the relationship between training and OCB Change at Transfer2

	Model A (Without Moderation)		Model B (With Moderation)	
	**Standardized Coefficients**	**t-value**	**Standardized Coefficients**	**t - value**
Step 1				
Training	.581***	5.799	.581***	5.761
Social and Organizational Support	-.177	-1.765	-.185	-1.627
Step2				
Training * Social and organizational support			-.018	-.161
R2 Change	.310		.000	
F Change	16.817***		.026	

Supervisor Evaluation Sample: Moderation Analysis on the relationship between training and CWB Change at Transfer2

	Model A (Without Moderation)	Model B (With Moderation)

	**Standardized Coefficients**	**t-value**	**Standardized Coefficients**	**t - value**
Step 1				
Training	-.339**	-2.982	-.339**	-2.964
Social and Organizational Support	.181	1.597	.202	1.561
Step2				
Training * Social and organisational support			.042	.336
R2 Change	.112		.001	
F Change	4.742*		.113	

Note: *p < 0.05, **p < 0.01, ***p < 0.001
